# Genome-Wide Identification and Expression Analysis of *NRAMP* Family Genes in Soybean (*Glycine Max* L.)

**DOI:** 10.3389/fpls.2017.01436

**Published:** 2017-08-18

**Authors:** Lu Qin, Peipei Han, Liyu Chen, Thomas C. Walk, Yinshui Li, Xiaojia Hu, Lihua Xie, Hong Liao, Xing Liao

**Affiliations:** ^1^Key Laboratory of Biology and Genetics Improvement of Oil Crops of the Ministry of Agriculture, Oil Crops Research Institute of Chinese Academy of Agricultural Sciences Wuhan, China; ^2^Root Biology Center, Fujian Agriculture and Forestry University Fuzhou, China; ^3^Golden Fidelity LLC St. Louis, MO, United States

**Keywords:** soybean, *NRAMP* gene family, nutrient deficiency, divalent metal toxicity, nodules

## Abstract

The *NRAMP* (natural resistance-associated macrophage protein) family of genes has been widely characterized in organisms ranging from bacteria to yeast, plants, mice, and humans. This gene family plays vital roles in divalent metal ion transport across cellular membranes. As yet, comprehensive analysis of *NRAMP* family genes has not been reported for soybean. In this study, bioinformatics analysis was conducted to identify 13 soybean *NRAMP* genes, along with their gene structures, phylogenetic relationships, and transmembrane domains. Expression analysis suggests that *GmNRAMP* genes function in numerous tissues and development stages. Moreover, soybean *NRAMP* genes were differentially regulated by deficiencies of N, P, K, Fe, and S, along with toxicities of Fe, Cu, Cd, and Mn. These results indicate that *GmNRAMP* genes function in many nutrient stress pathways, and might be involved in crosstalk among nutrient stress pathways. Subcellular localization analysis in Arabidopsis protoplasts confirmed the tonoplast or plasma membrane localization of selected soybean NRMAP proteins. Protein-protein interaction analysis found that the networks of three GmNRAMP proteins which putatively interact with nodulin-like proteins, almost distinct from the network that is common to the other 10 soybean NRAMP proteins. Subsequent qRT-PCR results confirmed that these three *GmNRMAP* genes exhibited enhanced expression in soybean nodules, suggesting potential functions in the transport of Fe or other metal ions in soybean nodules. Overall, the systematic analysis of the *GmNRAMP* gene family reported herein provides valuable information for further studies on the biological roles of *GmNRAMPs* in divalent metal ion transport in various soybean tissues under numerous nutrient stresses and soybean-rhizobia symbiosis.

## Introduction

Iron (Fe) is an essential element for plant development and growth, with functions in several basic cellular processes, including photosynthesis, respiration, and chlorophyll biosynthesis (Kobayashi and Nishizawa, 2012). Furthermore, Fe is also a vital component in heme, the Fe-sulfur (S) cluster, and other Fe-binding sites (Kobayashi and Nishizawa, 2012). Given these requirements and Fe deficiency is common in soils, plants have evolved highly efficient systems to acquire Fe from the soil (Kim and Guerinot, 2007). Furthermore, the uptake, utilization, and storage of Fe are also tightly controlled by the coordination of multiple mechanisms regulated to at the transcriptional and post-translational levels (Kobayashi and Nishizawa, 2012). Mechanisms contributing to Fe acquisition in a number of plant species can be divided into two categories (Hell and Stephan, 2003; Morrissey and Guerinot, 2009; Conte and Walker, 2011). Strategy I, which is found in non-graminaceous plants, utilizes *IRT1* as the primary transporter responsible for uptake of Fe from soil into roots (Eide et al., 1996; Hell and Stephan, 2003; Walker and Connolly, 2008). Meanwhile, YSL is the main transporter responsible for uptake of Fe from siderophore-Fe complexes into Strategy II graminaceous plants (Curie et al., 2001; Inoue et al., 2009; Thomine and Vert, 2013). Beyond these Strategy I and II transporters, the NRAMP family represents another transporter family associated with Fe uptake and transport (Thomine and Vert, 2013).

The NRAMP family, with its highly conserved domain, is widespread in genomes ranging from bacteria to humans (Nevo and Nelson, 2006). It is known to mediate transport of divalent metal ions, such as Fe and manganese (Mn) across cellular membranes. The first known NRAMP protein (NRAMP1) was discovered in mice phagosomal membranes, and was found to function in natural defense against infections by intracellular parasites (Vidal et al., 1993). In contrast to NRAMP1, mice NRAMP2 (also called DMT1), yet it still acts as a divalent metal ion transporter in the absorption of Fe, Mn, zinc (Zn), copper (Cu), cadmium (Cd), and lead (Pb) (Garrick et al., 2006). Mutations in *NRAMP2* have been associated with defects in Fe absorption and result in Microcytic anemia in mice and the anemic Belgrade rat (Fleming et al., 1998). NRAMP homologs with similar function also were found in human (Cellier et al., 1994; Beaumont et al., 2006; Illing et al., 2012).

Several *NRAMP* gene family members have also been functionally characterized in plants. In Arabidopsis, there are six NRAMP proteins (Mäser et al., 2001). AtNRAMP1 regulates Fe homoeostasis (Curie et al., 2000), and function as a high-affinity transporter for Mn uptake (Cailliatte et al., 2010). AtNRAMP3 and AtNRAMP4 are both localized on the vacuolar membrane and participate in vacuolar Fe mobilization during seed germination (Lanquar et al., 2005). AtNRAMP6 is targeted to a vesicular-shaped endomembrane compartment and functions as an intracellular metal transporter, with possible involvement in Cd tolerance (Cailliatte et al., 2009). In rice, three NRAMP proteins participate in Fe, Cd, and Mn uptake (Takahashi et al., 2011; Sasaki et al., 2012; Yang et al., 2014), while another, OsNrat1, encodes a transporter mediating aluminum (Al) uptake from root tip cell walls into the cell, which contributes to rice Al tolerance (Li et al., 2014). In recent years, several *NRAMP* genes have been identified in legumes. For example, a peanut *NRAMP* gene, *AhNRAMP1*, is significantly induced by Fe deficiency in roots and leaves, and heterologous expression of *AhNRAMP1* in tobacco leads to Fe accumulation in young leaves and tolerance to Fe deprivation (Xiong et al., 2012). Moreover, in the model legume *Medicago truncatula, MtNRAMP1*, is mainly localized to the plasma membrane, with expression levels highest in roots and nodules, suggesting it was the major transporter responsible for apoplastic Fe uptake in rhizobia-infected cells (Tejada-Jiménez et al., 2015).

Provided the commercial significance of Soybean (*Glycine max* L.) worldwide and the detrimental effects of Fe deficiency on yield and quality, it is key to improve our understanding of Fe transport as tool for improving soybean Fe utilization. However, little data is available concerning the *NRAMP* gene family in soybean until now. In the present study, bioinformatics analysis was conducted to identify 13 soybean *NRAMP* genes. Subsequently, tissue-specific expression of *GmNRAMP* genes and their responses to various nutrient stresses were all analyzed. The genome-wide analysis of soybean *NRAMP* genes herein provides a basis to further investigate detailed functions of *NRAMP* genes in soybean.

## Materials and methods

### Identification and bioinformatics analyses of *NRAMP* genes in soybean

To identify NRAMP homologs in soybean, nucleic acid, and amino sequences of all reported NRAMPs in Arabidopsis, Rice and Medicago, were used as query sequences in BLASTN (Target type: Genome) and BLASTP (Target type: Proteome) searches of the *G. max* cultivar Williams 82 in the Phytozome genome database (https://phytozome.jgi.doe.gov/pz/portal.html#) using default settings for E-value and the number of hit sequences. Then, all returned genes and proteins were further examined for inclusion of the conserved Nramp domain (PF01566) by querying in the Uniprot (http://www.uniprot.org/) and Pfam (http://pfam.xfam.org/search) databases. The nucleic acid and amino sequences of identified soybean *NRAMP* genes were downloaded from the Phytozome website. Soybean *NRAMP* genes were named according to phylogenetic relationships among the proteins. The chromosomal localization map and duplication of soybean *NRAMP* gene was determined by using ORTHOMCL (http://orthomcl.org/orthomcl/) and SVG softwares (http://search.cpan.org/~ronan/SVG-2.28/SVG/Manual.pm). Protein molecular weights and theoretical *pI* values were computed using ProtParam (http://web.expasy.org/protparam/). Sequence identity among soybean NRAMP proteins was determined using BLASTP with each sequence queried against the other soybean NRAMP sequences in standalone BLAST downloaded from NCBI (https://blast.ncbi.nlm.nih.gov/Blast.cgi). Transmembrane helices in proteins were predicted using the TMHMM Server v. 2.0 (http://www.cbs.dtu.dk/services/TMHMM/). Predictions of subcellular localization for soybean NRAMP proteins were generated with ProtComp 9.0 (http://linux1.softberry.com/berry.phtml?group=programs&subgroup=proloc&topic=protcomppl). Multiple sequence alignment was performed with Clustal W and drawn in Genedoc, with the logo of consensus transport residues then generated by WebLogo 3 (http://weblogo.threeplusone.com/). Phylogenetic trees based on full length protein sequence alignments of NRAMPs from soybean and several other species were constructed by the neighbor-joining method with 1,000 bootstrap replicates in MEGA 6.0 (http://www.megasoftware.net/download_form). Downloaded CDS and genomic sequences of soybean *NRMAP* genes were used to construct gene structures on the Gene Structure Display Server 2.0 (http://gsds.cbi.pku.edu.cn/index.php). PlantCARE (http://bioinformatics.psb.ugent.be/webtools/plantcare/html/) was used to *cis*-element analysis in the 1,500 bp region upstream of the start codon for each *NRAMP* gene.

### Plant materials and treatments

Soybean cv. Williams 82 was employed in this study. For tissue specific expression analysis of *GmNRAMPs*, soybean plants were cultured in hydroponics and harvested at a number of developmental stages for qRT-PCR assays. Specifically, soybean seeds were surface-sterilized in 10% H_2_O_2_, then, after germination for 1 week, seedlings were transplanted into full-strength nutrient solution as previously described (Li et al., 2012) which contained 250 mM KH_2_PO_4_, 3,000 mM KNO_3_, 2,000 mM Ca(NO_3_)_2_, 250 mM MgSO_4_, 25 mM MgCl_2_, 12.5 mM H_3_BO_3_, 1 mM MnSO_4_, 1 mM ZnSO_4_, 0.25 mM CuSO_4_, 0.25 mM (NH_4_)_6_Mo_7_O_24_, and 25 mM Fe-Na-EDTA. The pH value of the nutrient solution was adjusted to 5.8, and nutrient solution was changed weekly. Seedlings were grown in a growth chamber with a 16 h light and 8 h dark cycle at 28°C for 40 days before separately harvesting young leaves, older leaves, roots, stems, and flowers. At 55 days, young pods and seeds were also separately harvested. All tissue samples were stored at −80°C for further RNA extraction and qRT-PCR analysis.

To investigate possible responses of *GmNRAMPs* to nutrient deficiency, 10-day-old seedlings were exposed to low-nitrogen (LN), -phosphorus (LP), -potassium (LK), -iron (LFe), or -sulfur (LS) conditions for 14 days, in which time nutrient deficiency symptoms became evident. For the LN treatment, KNO_3_ and Ca(NO_3_)_2_ were replaced by K_2_SO_4_ and CaCl_2_, respectively. For the LP treatment, KH_2_PO_4_ was replaced by K_2_SO_4_. For LK, KNO_3_, and KH_2_PO_4_ were replaced by Ca(NO_3_)_2_ and NaH_2_PO_4_, respectively. For LFe, Fe-Na-EDTA was not added to the nutrient solution. In the LS treatment, MgSO_4_ was replaced by MgCl_2_. Seedlings grown in full-strength nutrient solution were sampled as the control. Each treatment had four biological replicates. Leaves and roots were separately sampled for further RNA extraction and qRT-PCR analysis.

To elucidate the probable functions of *GmNRAMPs* in response to divalent metal toxicity stresses, 10-day-old seedlings were treated with excess Fe (1,000 μM EDTA-Fe), Cu (200 μM CuSO_4_·5H_2_O), Cd (100 μM CdCl_2_), and Mn (200 μM MnSO_4_·H_2_O) treatments for 24 h. Each treatment had four biological replicates. Leaves and roots were separately sampled for further analysis.

To further study the responses of *GmNRAMPs* to rhizobia inoculation, 7-day-old seedlings were inoculated with the effective rhizobial strain Bradyrhizobium sp. BXYD3 (Cheng et al., 2009), and then transplanted into low nitrogen (500 μM N added) nutrient solution. Each treatment had four biological replicates. Young leaves, stems, roots, and nodules were separately collected at 30 days after inoculation and then stored at –80°C for RNA extraction and qRT-PCR analysis.

### RNA extraction and qRT-PCR analysis

Total RNA was extracted from different soybean samples using RNAiso™ Plus reagent (Takara Bio, Otsu, Shiga, Japan) according to the manufacturer's instructions. RNA samples were treated with RNase-free DNaseI (Invitrogen, Grand Island, NY, USA) to remove contaminating genomic DNA. The quality of total RNA was checked via spectrophotometry (TGem Plus, Tiangen, China). Then, first strand cDNA was synthesized using the PrimeScript™ RT Master Mix (Takara, Tokyo, Japan) according to the manufacturer's protocol. For qRT-PCR analysis, the soybean housekeeping gene *TefS1* (encoding the elongation factor EF-1a: X56856) was used as a reference gene, and specific primers for *GmNRAMPs* and *TefS1* were designed with Primer-NCBI (https://www.ncbi.nlm.nih.gov/tools/primer-blast/index.cgi?LINK_LOC=BlastHome) as listed in Table S1. In addition, the specific primers for nutrient deficiency responsive genes were also designed and listed in Table S2. qRT-PCR reactions were carried out in a CFX connect Real-Time PCR Detection System (Bio-Rad, Hercules, USA) with SYBR® Premix Ex Taq™ II (TaKaRa, Tokyo, Japan). The PCR reaction volume was 20 μL in total, which included 2 μL diluted cDNA, 10 μL SYBR Premix Ex Taq™ reagent, 0.6 μL primers and 6.8 μL RNA-free water. PCR Reactions were performed under the following conditions: 95°C for 1 min, followed by 40 cycles of 95°C for 15 s, 60°C for 15 s, and 72°C for 30 s. The expression of *NRAMP* genes was calculated by the 2^−Δ^Cq and 2^−ΔΔ^Cq methods (Livak and Schmittgen, 2001).

### Subcellular localization and predicted protein interaction networks

To determine the predicted subcellular localization of soybean NRAMP proteins, six GmNRAMP proteins were selected to generate subcellular localization constructs, then transient expression in Arabidopsis protoplasts, which were widely used in subcellular localization analysis of genes not only for soybean, but also for other plant species (Zhang et al., 2016; Chong et al., 2017; Chu et al., 2017; Péron et al., 2017; Xu et al., 2017). Specifically, the coding region of each *GmNRAMP* gene was amplified with gene-specific primers shown in Table S3. These CDS sequences were cloned into the pMDC43 vector to express GmNRAMP-GFP fusion proteins driven by the CaMV 35S promoter. The constructs of 35S::GmNRAMP-GFP and 35S:GFP (control) were separately transformed into Arabidopsis protoplasts. Arabidopsis protoplasts were isolated from the leaves of 4-week-old Arabidopsis plants and subsequently transformed according to previously published protocols (Yoo et al., 2007). After transfection using polyethylene glycol and incubation in a plate under weak light for 12–16 h, protoplasts were observed with an Olympus FV10-ASW laser scanning confocal microscope (Olympus, Japan). Corresponding markers used in coexpression experiment were selected according to predicted subcellular localizations of GmNRAMP proteins, which were also verified in rice protoplasts isolated from the stems of 10-day-old rice plants under dark culture conditions. The coexpression of two marker genes in rice protoplasts were performed same as the protocol for Arabidopsis protoplasts.

To further investigate possible protein interactions involving GmNRAMPs, putative interaction networks were identified in the interaction section of the UniProt protein knowledgebase (http://www.uniprot.org/), with interactions originating from STRING 10.0 protein-protein interaction databases (http://www.string-db.org/). The default settings of association networks were applied in these analyses.

### Statistical analysis

All data were analyzed using Microsoft Excel 2010 (Microsoft Company, USA) for calculating mean and standard error. Comparisons of gene expression among genes and tissues using analysis of variance (ANOVA) and Duncan's Multiple Range Test (DMRT) for mean separation, as well as, in response to nutrient deficiencies using *t*-tests were performed in RStudio (RStudio, USA) using standard R packages (https://www.R-project.org). Resulting *p*-values from *t*-tests were corrected for false discovery in multiple hypotheses testing by manually calculating adjusted p (Q) values in Excel using the Benjamini-Hochberg method (Benjamini and Hochberg, 1995). Comparisons of gene expression in response to metal toxicity and rhizobia inoculation were also performed using analysis of variance (ANOVA) in Excel.

## Results

### Genome-wide identification and bioinformatic analysis of soybean *NRAMP* family genes

**Figure 1:**
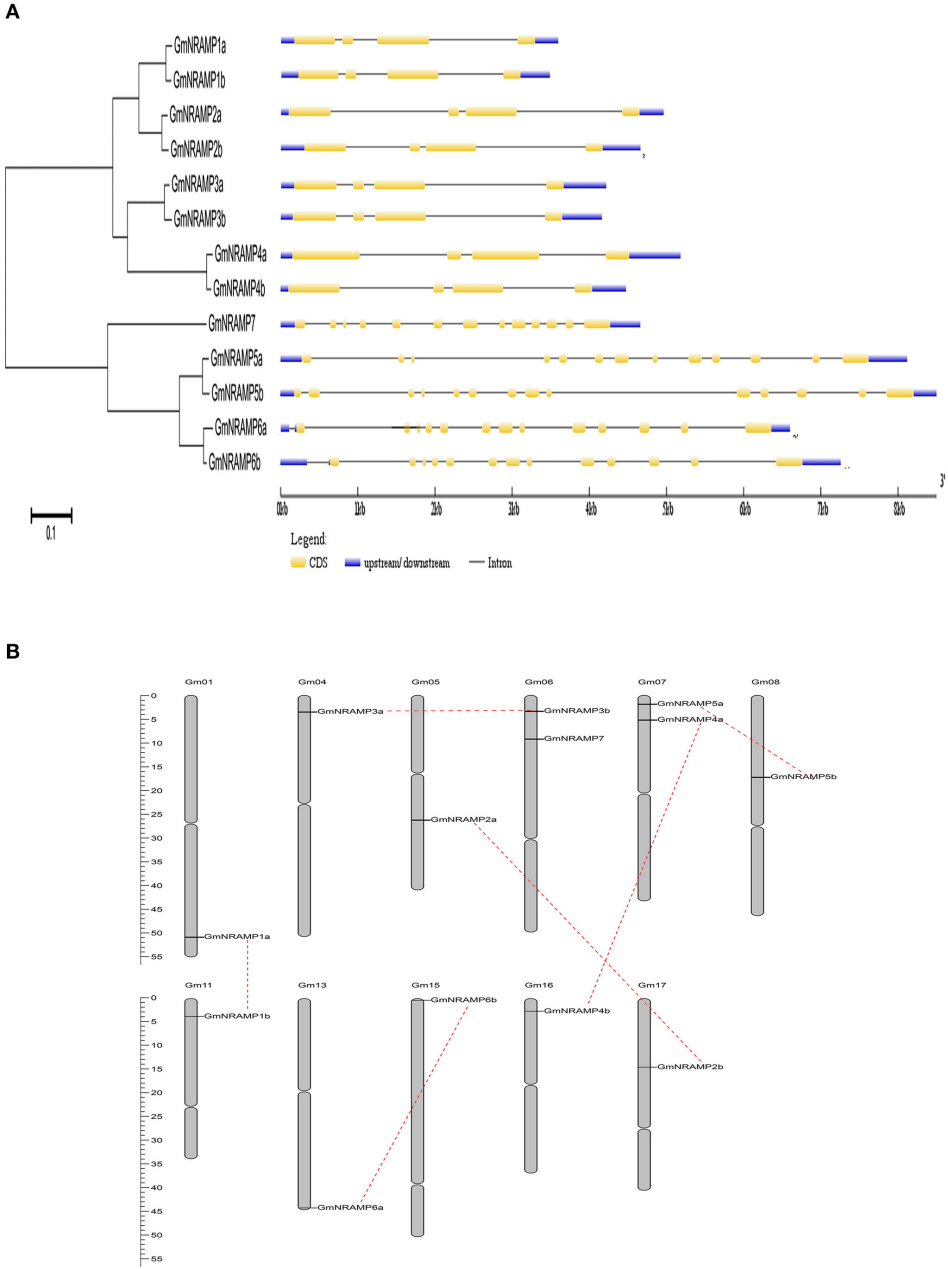
Phylogenetic relationships, gene structures, and chromosomal positions of soybean *NRAMP* family genes. **(A)** Phylogenetic relationships of soybean *NRAMP* family genes. The phylogenetic tree was constructed based on sequence alignment of soybean NRAMPs using the neighbor-joining method with bootstrapping analysis in MEGA 6.0 (1,000 replicates). Gene structures were drawn by Gene Structure Display Server 2.0 with genomic sequences and CDS sequences of each *NRAMP* gene. Introns and exons are represented by black lines and yellow boxes, respectively. Blue boxes represent UTRs. **(B)** Positions of *NRAMP* family genes on soybean chromosomes.

Thirteen putative *GmNRAMP* genes were identified in BLAST searches of the *G. max* cv. Williams 82 reference genome in the Phytozome database using arabidopsis, rice, and medicago NRAMP as query sequences. All identified *GmNRAMPs* were named based on phylogenetic relationships among soybean NRAMP family members (Figure 1A and Table 1), with the tree being comprised of two main branches (Figure 1A). The NRAMP family domain (PF01566) and 10–12 putative transmembrane domains (TMDs) were also found in each putative GmNRAMP protein (Table 1 and Figure S1). The CDS regions of putative *GmNRAMP* genes range in length from 1,521 to 1,767 bp and encode proteins with lengths of 506–588 amino acid residues, molecular weights of 55.44–64.39 KDa, and *pI* values of 4.77–9.04 (Table 1). Gene structures were similar within each of the two main subfamilies as illustrated in Figure 1A. Subfamily I is comprised of 8 members, each with 4 exons and 3 introns, while the five Subfamily II GmNRAMPs each have 13–14 exons and 12–13 introns. Among the Subfamily II proteins, GmNRAMP7, with a relatively short length, appears to be divergent from the other four members. It is also worth mentioning that GmNRAMP6a and GmNRAMP6b each contain 13 introns, with one intron located in the 5′ UTR (Figure 1A). Chromosome mapping showed that the 13 *GmNRAMPs* are distributed on 11 chromosomes. Chromosomes 6 and 7 each contained two *NRAMPs*, while chromosomes 1, 4, 5, 8, 11, 13, 15, 16, and 17 each contained one *NRAMP* (Figure 1B). From the phylogenetic tree of soybean NRAMP proteins, we noticed that all but GmNRAMP7 appeared in pairs, implying possible gene duplication occurred during evolution of *NRAMP* gene family, therefore, synteny analysis also performed to determine the potential gene duplication with soybean *NRAMP* family. As shown in Figure 1B, six pairs of soybean *NRAMP* genes were found to be located in duplicated blocks, suggesting the duplication event also happened in the soybean *NRAMP* gene family.

**Table 1:** Summary of *NRAMP* family genes in soybean.

**Gene name**	**Gene locus**	**Length of CDS (bp)**	**No. of amino acids (aa)**	**MW (KD)**	**PI**	**Protein domain family**	**Prediction of subcellular localization**
*GmNRAMP1a*	Glyma.01G190700	1,524	507	55.64	5.33	NRAMP (PF01566)	Vacuole
*GmNRAMP1b*	Glyma.11G051500	1,521	506	55.44	5.07	NRAMP (PF01566)	Vacuole
*GmNRAMP2a*	Glyma.05G101700	1,551	516	56.58	5.23	NRAMP (PF01566)	Vacuole
*GmNRAMP2b*	Glyma.17G165200	1,551	516	56.59	4.98	NRAMP (PF01566)	Vacuole
*GmNRAMP3a*	Glyma.04G044000	1,557	518	56.73	5.20	NRAMP (PF01566)	Vacuole
*GmNRAMP3b*	Glyma.06G044200	1,569	522	57.37	5.12	NRAMP (PF01566)	Vacuole
*GmNRAMP4a*	Glyma.07G058900	1,674	557	61.76	4.86	NRAMP (PF01566)	Vacuole
*GmNRAMP4b*	Glyma.16G027800	1,680	559	61.99	4.77	NRAMP (PF01566)	Vacuole
*GmNRAMP5a*	Glyma.07G023600	1,638	545	59.17	8.50	NRAMP (PF01566)	Plasma membrane
*GmNRAMP5b*	Glyma.08G218200	1,767	588	64.39	9.04	NRAMP (PF01566)	Plasma membrane
*GmNRAMP6a*	Glyma.13G369900	1,635	544	59.07	8.46	NRAMP (PF01566)	Plasma membrane
*GmNRAMP6b*	Glyma.15G003500	1,641	546	59.20	8.74	NRAMP (PF01566)	Plasma membrane
*GmNRAMP7*	Glyma.06G115800	1,635	544	58.65	8.86	NRAMP (PF01566)	Plasma membrane

**Figure 2:**
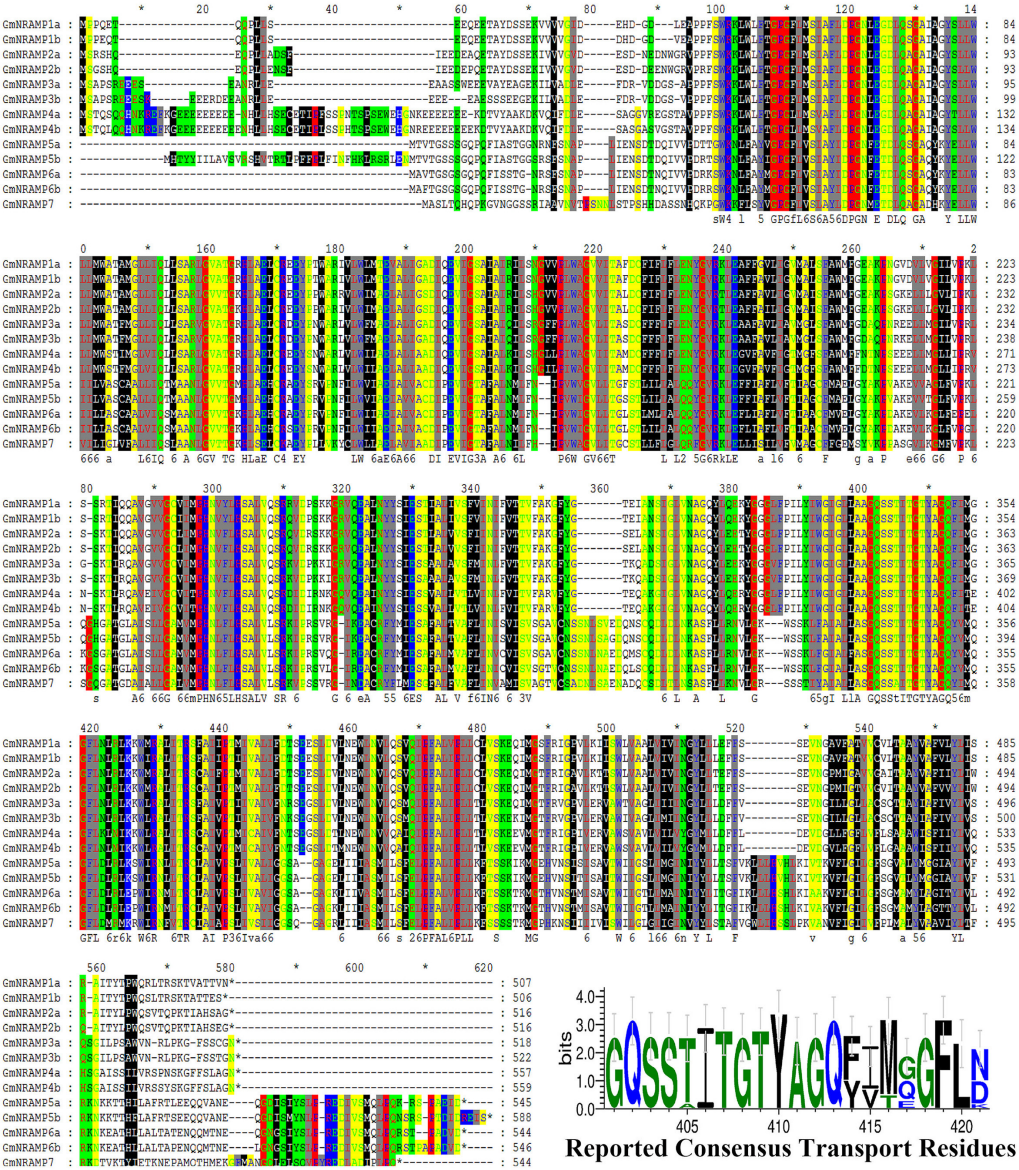
Multiple alignment of soybean NRAMP family proteins. Multiple alignment was performed with Clustal W and the residues were colored using Genedoc software. Red lines underneath alignments indicate reported consensus transport residues. “^*^” above the sequence mean every ten amino acid residues. The logo of these residues was then generated in WebLogo 3 online.

In BLASTP analysis, all alignments included at least 73% of each GmNRAMP sequence, while 102 of the 169 alignments incorporated over 90% of the protein sequence (data not shown). Sequence identity in aligned regions ranged from 38 to 98% outside of self hits (Table 2), with the highest percentage of identity occurring between GmNRAMP3a and GmNRAMP3b which also fell very close to each other in the phylogenetic tree (Figure 1A). The lowest identity, on the other hand, occurred between GmNRAMP4b and GmNRAMP7 (Table 2). The reported consensus transport residues, GQSSTITGTYAGQY(/F)V(/I)MQGFLD(/E/N) were present in all identified soybean NRAMP sequences (Figure 2).

**Table 2:** Percentage of protein sequence identity in BLASTP alignments among the 13 soybean NRAMP proteins.

**% ident.**	**NRAMP1a (%)**	**NRAMP1b (%)**	**NRAMP2a (%)**	**NRAMP2b (%)**	**NRAMP3a (%)**	**NRAMP3b (%)**	**NRAMP4a (%)**	**NRAMP4b (%)**	**NRAMP5a (%)**	**NRAMP5b (%)**	**NRAMP6a (%)**	**NRAMP6b (%)**	**NRAMP7 (%)**
NRAMP1a	100												
NRAMP1b	97	100											
NRAMP2a	82	83	100										
NRAMP2b	83	84	96	100									
NRAMP3a	78	79	78	78	100								
NRAMP3b	80	80	79	78	98	100							
NRAMP4a	68	67	69	69	73	71	100						
NRAMP4b	68	68	69	69	70	73	96	100					
NRAMP5a	43	44	43	44	42	43	39	39	100				
NRAMP5b	44	44	45	42	42	43	39	39	96	100			
NRAMP6a	39	39	41	38	41	42	39	40	85	84	100		
NRAMP6b	44	43	44	42	41	42	39	40	85	84	97	100	
NRAMP7	39	40	40	40	39	39	39	38	63	61	58	59	100

**Figure 3:**
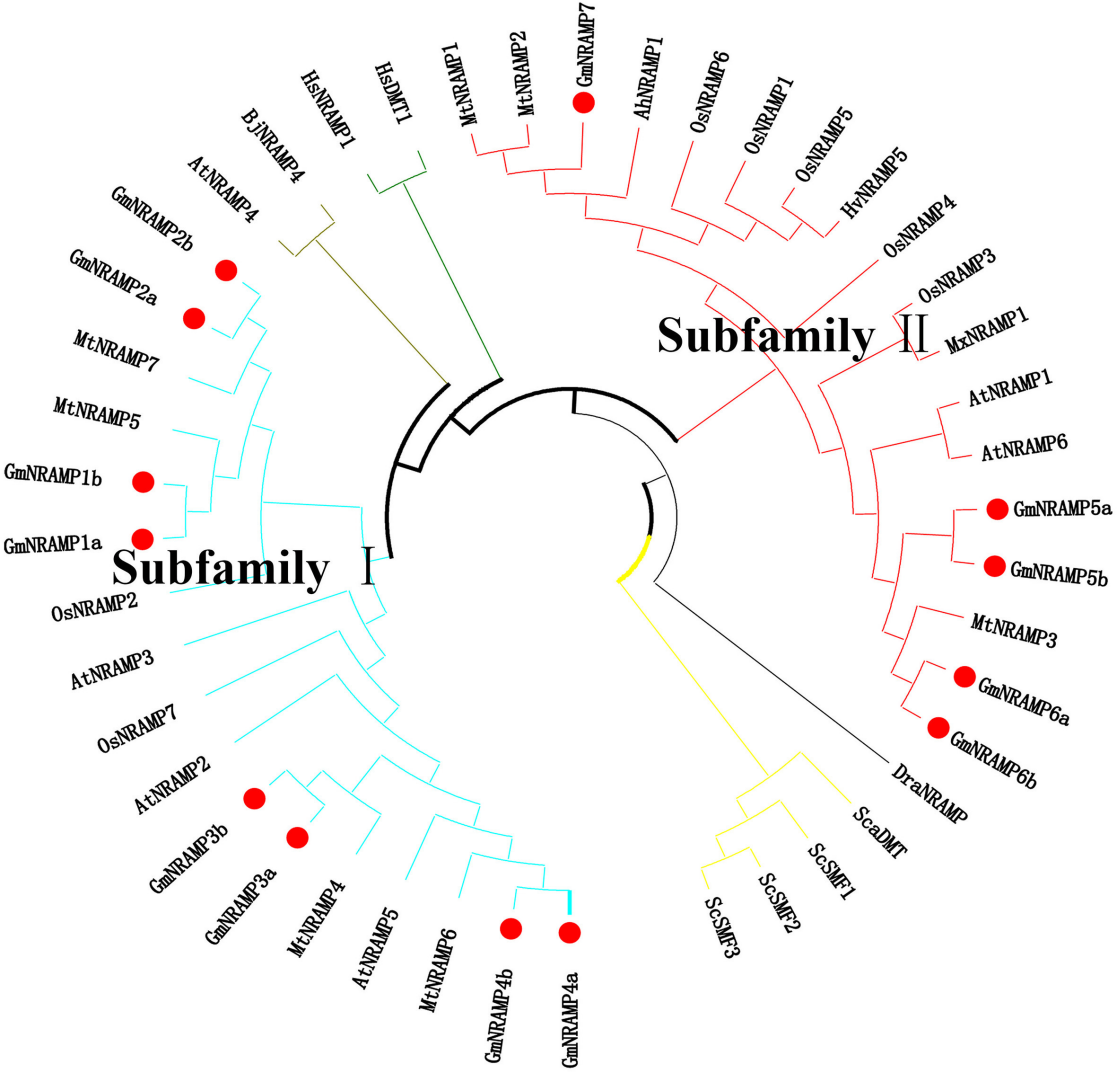
Phylogenetic relationship of *NRAMP* family genes from soybean and other species. The phylogenetic tree was constructed based on sequence alignment of NRAMP homologs from Soybean (Gm), Arabidopsis (At), Rice (Os), Medicago (Mt), Barley (Hv), Peanut (Ah), Apple (Mx), Mustard (Bj), Yeast (Sc and Sca), *Deinococcus radiodurans* (Dra), and Human (Hs) using the neighbor-joining method with bootstrapping analysis in MEGA 6.0 (1,000 replicates). The 44 included NRAMP proteins from 11 species are divided into different branches with different color labels. Plant NRAMP proteins, with one exception cluster into two groups (subfamily I and subfamily II). Soybean NRAMP proteins are shown in bold with red filled circles.

Further phylogenetic investigation was conducted with the inclusion of NRAMPs from other plant species, bacteria, fungi and humans. As expected (Figure 3), NRAMPs from *Deinococcus radiodurans* (DraNRAMP), *Staphylococcus capitis* (ScaDMT), and *Saccharomyces cerevisiae* (ScSMF1, ScSMF2, and ScSMF3) were closely related and separated from higher plant and human NRAMPs (HsDMT1 and HsNRAMP1). The tested higher plant NRAMPs from soybean, arabidopsis, rice, medicago, barley, peanut, apple, and mustard, with one exception, fall into two large groups, subfamily I and subfamily II, with both monocots and dicots represented in each of these two groups. Arabidopsis carries four subfamily I members and two subfamily II members, while the corresponding subfamily I and II numbers were two and five for rice, and four and three from medicago. The 13 soybean NRAMP members sorted into eight subfamily I members and five subfamily II members, which matched the phylogenic and gene structure results described above. In addition, the tested NRAMP proteins from mustard clustered into subfamily I, while the NRAMP proteins from peanut, barley, and apple clustered into subfamily II (Figure 3). Interestingly, the subcellular localization predictions for GmNRAMPs indicate that subfamily I members localize to vacuoles, while subfamily II members localize to the plasma membrane (Table 1). To better understand the potential regulation of *GmNRAMP* genes, *cis*-element analysis was performed and shown in Table S4. A number of *cis-acting* regulatory elements involved in light responsiveness, were frequently identified in soybean *NRAMP* genes promoter regions (Table S4). In addition, *cis*-regulatory elements in *GmNRAMPs* were also associated with various stress factors. Notably, the promoter regions of 9 *NRAMP* genes contained *cis*-elements related to defense and stress responsiveness (Table S4). Most *NRAMP* gene promoters contained at least one HSE element (heat stress responsiveness), which was followed in prevalence by MES (drought-inducible) and LTR (low temperature responsiveness) elements. Furthermore, several identified elements in *GmNRAMP* promoter regions have been reported to be involved in hormone responsiveness. Specifically, all *GmNRAMPs* except *GmNRAMP6b* were associated with the *cis*-acting element ABRE, which is involved in abscisic acid (ABA) responsiveness. Plus, the *GmNRAMP2a* and *GmNRAMP4b* promoter regions contained a series of elements responsive to nearly all types of hormone, including ABA, Methyl Jasmonate (MeJA), ethylene (ETH), gibberellin (GA), and auxin (IAA). These results indicate that *GmNRAMP* genes may be involved in complex regulatory networks and could be regulated by various environmental, developmental, and physiological factors.

### Tissue-specific expression of *GmNRAMPs*

**Figure 4:**
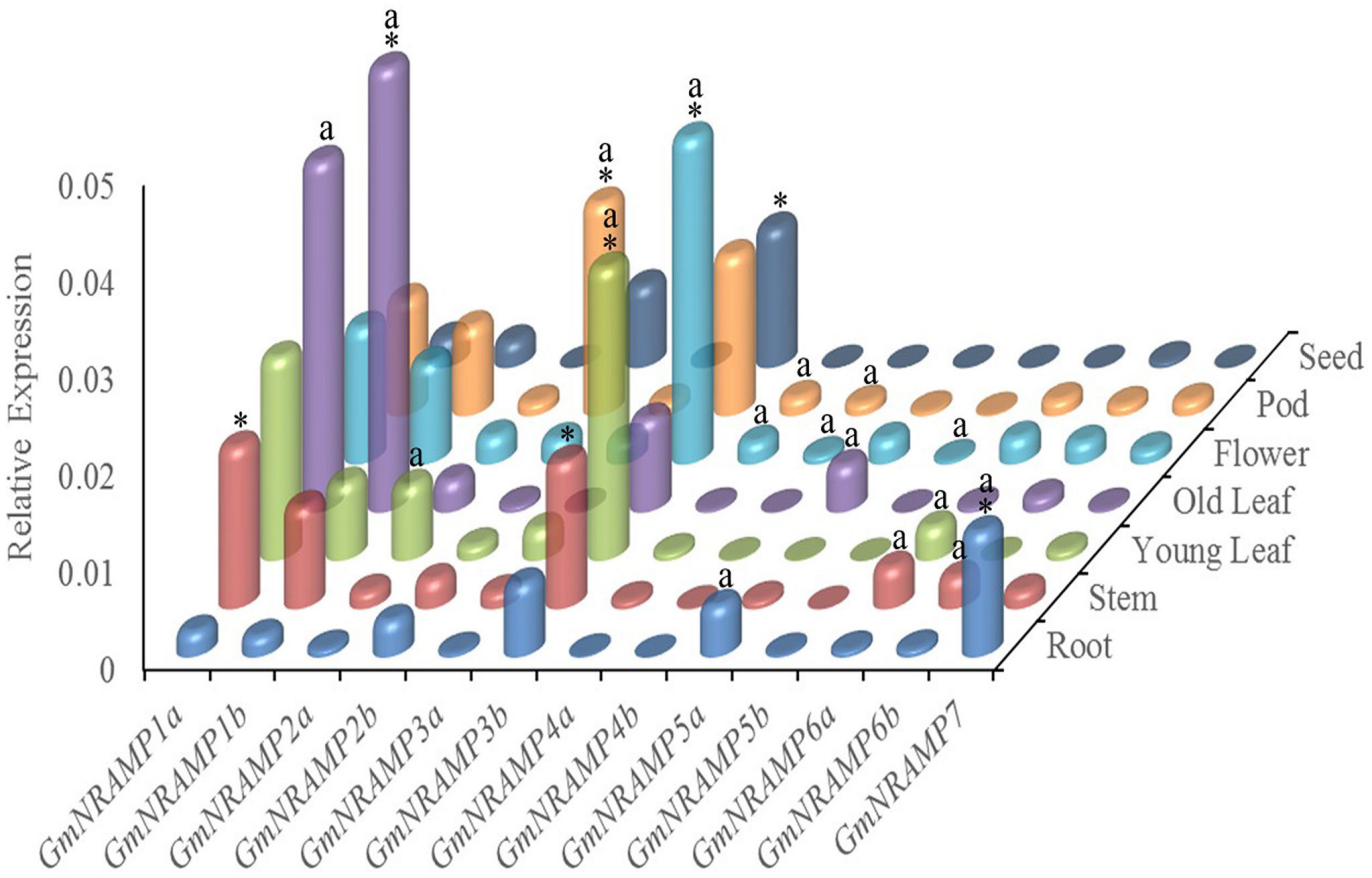
Tissue-specific expression profiles of *GmNRAMP* genes. Quantitative RT-PCR analysis of *GmNRAMP* genes was performed in various soybean tissues. Soybean seedlings were grown for 40 days in hydroponic cultures prior to separately harvesting young leaves (YL), older leaves (OL), stems (ST), roots (R), and flowers (F). Then, at 55 days, young pods (P) and seeds (SE) were separately harvested. Relative expression levels were obtained by real-time PCR normalized against the reference gene *TefS1*. Each bar is the mean of four biological replicates with standard error. Differential expression was tested among tissues for each gene, and among genes for each tissue by ANOVA tests followed by the Duncan's Multiple Range Test for mean separation. The letter “a” marks tissues in which each gene is most highly expressed, and an “^*^” marks the genes that are most highly expressed in each tissue.

In order to investigate tissue-specific expression profiles of *GmNRAMPs*, qRT-PCR analysis was performed with seven soybean tissues, namely young leaves, older leaves, stems, roots, flowers, pods, and seeds. Using an ANOVA significance threshold of *p* = 0.05, expression varied among tissues for each of the 13 tested *GmNRAMPs*, as well as, among *GmNRAMP* genes within each tissue. Quantified expression levels for each *GmNRAMP* gene in each of the seven tissues are displayed in Figure 4, with those in the most highly expressed category according to Duncan's Multiple Range Test marked by an “a” for the tissues in which each gene was most highly expressed, and an “^*^” for the most highly expressed *GmNRAMPs* in each tissue. Where *GmNRAMP* transcription was above the detection limit, expression levels varied by over 50-fold among tissues and *GmNRAMP* genes. Expression was low for *GmNRAMP*s *4b* and *5b* in all tissues, but was highest for both of these genes in flowers, as well as in pods for *GmNRAMP4b*. All *GmNRAMP*s were detected in each tissue, except seeds. The most highly expressed *GmNRAMP* in each tissue was *GmNRAMP*s *1a, 1b, 2b, 3b*, or *7*, with *GmNRAMP*s *1a, 1b, 2b*, and *3b* being notable for relatively high expression in multiple tissues. For leaves, relatively high expression was also observed for *GmNRAMP*s *2a, 3a*, and *6a* in young leaves, and for *GmNRAMP 5a* in old leaves. Higher expression was also observed for *GmNRAMP*s *6a* and *6b* in stems, and for *GmNRAMP 5a* in roots. In flowers and pods, appreciable expression was observed for all *GmNRAMP*s, except for *5a* and *5b* in pods. One notable result was that the most highly expressed *GmNRAMP*s typically belonged to subfamily II in all tissues, except in roots, where the subfamily I *GmNRAMP 7* was the most highly expressed *GmNRAMP*. Taken together, structures, phylogenies (Figure 1) and expression patterns (Figure 4) demonstrate that structurally similar *GmNRAMPs* also exhibit similar expression patterns.

### Expression of *GmNRAMPs* in response to macronutrient deficiency

**Figure 5:**
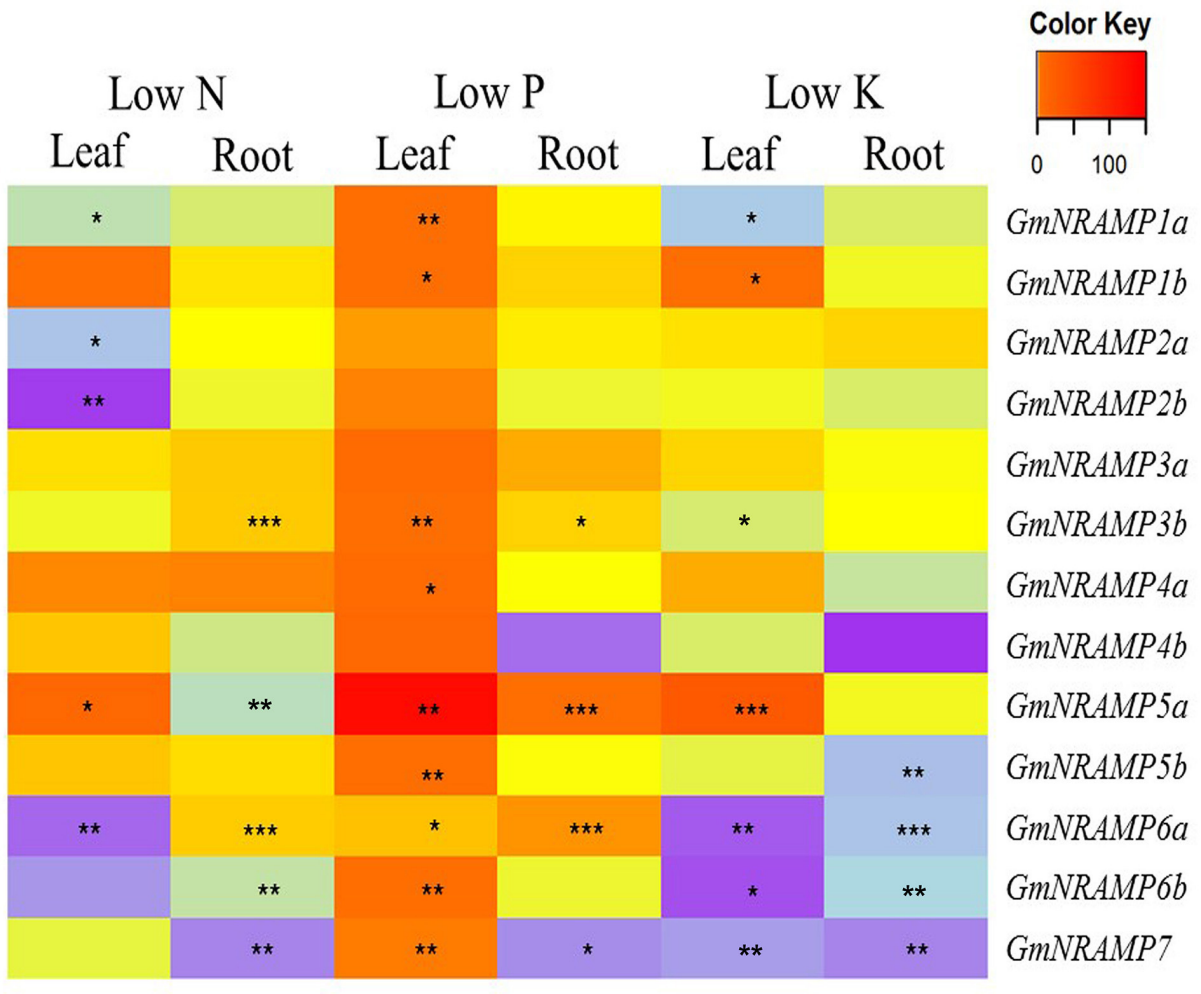
Expression profiles of *GmNRAMP* genes under nitrogen, phosphorus or potassium deficiency conditions. Ten-day-old soybean seedlings were subjected to nitrogen (LN), phosphorous (LP), or potassium (LK) deficiency conditions for 14 days, during which time obvious nutrient deficiency symptoms developed. Leaves and roots were separately harvested for qRT-PCR analysis. Fold-changes of *GmNRAMP* gene expression were normalized against the reference gene *TefS1* using 2^−ΔCt^ values. Differential expression was determined for four biological replicates in FDR adjusted *t*-tests of 2^−ΔCt^ values from nutrient deficient tissues vs. corresponding control tissues. The heat map displays 2^−ΔΔCt^ values to show relative expression of nutrient deficient samples vs. controls, with significant differences between treatment and control marked as follows, ^*^0.01 < *P* < 0.05, ^**^0.001 < *P* < 0.01, ^***^P < 0.001.

To evaluate potential responses of *GmNRAMPs* to macronutrient deficiencies, expression was assessed by qRT-PCR in soybean plants exposed to deficiencies of nitrogen (N), phosphorus (P), or potassium (K). *GmNiR*, which was repressed by N deficiency (Qin et al., 2012), together with *GmPLDZ* (a low P responsive gene) and *GmHAK* (a low K responsive gene), which had been demonstrated to respectively enhanced by P or K deficiency (Qin et al., 2012), were used to verify the nutrient deficiency treatments in this study (Figures S2A–C). Significant effects of macronutrient deficiencies were determined using FDR corrected *t*-tests for each *GmNRAMP* within each tissue for each nutrient treatment. As shown in Figure 5, the ratio of expression in deficient conditions relative to sufficient conditions significantly varied from constant expression for each *GmNRAMP* in one or more conditions and tissues. More specifically, N deficiency led to decreased expression of *GmNRAMP*s *1a, 2a, 2b*, and *6a* in leaves, and *GmNRAMP*s *5a, 6b*, and *7* in roots, while *GmNRAMP5a* was up-regulated in leaves, and *GmNRAMP*s *3b* and *6a* were up-regulated in roots. Under P deficiency conditions, *GmNRAMP* expression was dramatically altered. Nine *GmNRAMP*s, *1a, 1b, 3b, 4a, 5a, 5b, 6a, 6b*, and *7*, were up-regulated by P deficiency in leaves. In roots, *GmNRAMP*s *3b, 5a*, and *6a* were up-regulated, while *GmNRAMP7* was down-regulated. In comparison to N and P deficiency responses, K deficiency resulted primarily in decreased expression of *GmNRAMP* genes. *GmNRAMP*s *6a, 6b*, and *7* were down-regulated in both leaves and roots, while *GmNRAMP5b* was down-regulated in roots, and, in leaves, *GmNRAMP*s *1a* and *3b* were down-regulated and *GmNRAMP*s *1b* and *5a* were up-regulated. Among treatments and tissues, a few *GmNRAMP*s exhibited more notable responses. *GmNRAMP*s *5a, 6a*, and *7* responded to all treatments, except for *GmNRAMP7* in low N leaves and *GmNRAMP5a* in low K roots. Furthermore, *GmNRAMP7* responses were in the negative direction, except in low P leaves, while *GmNRAMP5a* responses were in the positive direction, except in low N roots. Two *GmNRAMP*s, *3a* and *4b*, displayed considerable variation in relative expression among tissues and nutrient treatments (Figure 5), yet this variation was not significant due to the overall low level of expression for each of these genes (Figure 4).

### Expression of *GmNRAMPs* in response to iron or sulfur deficiency

**Figure 6:**
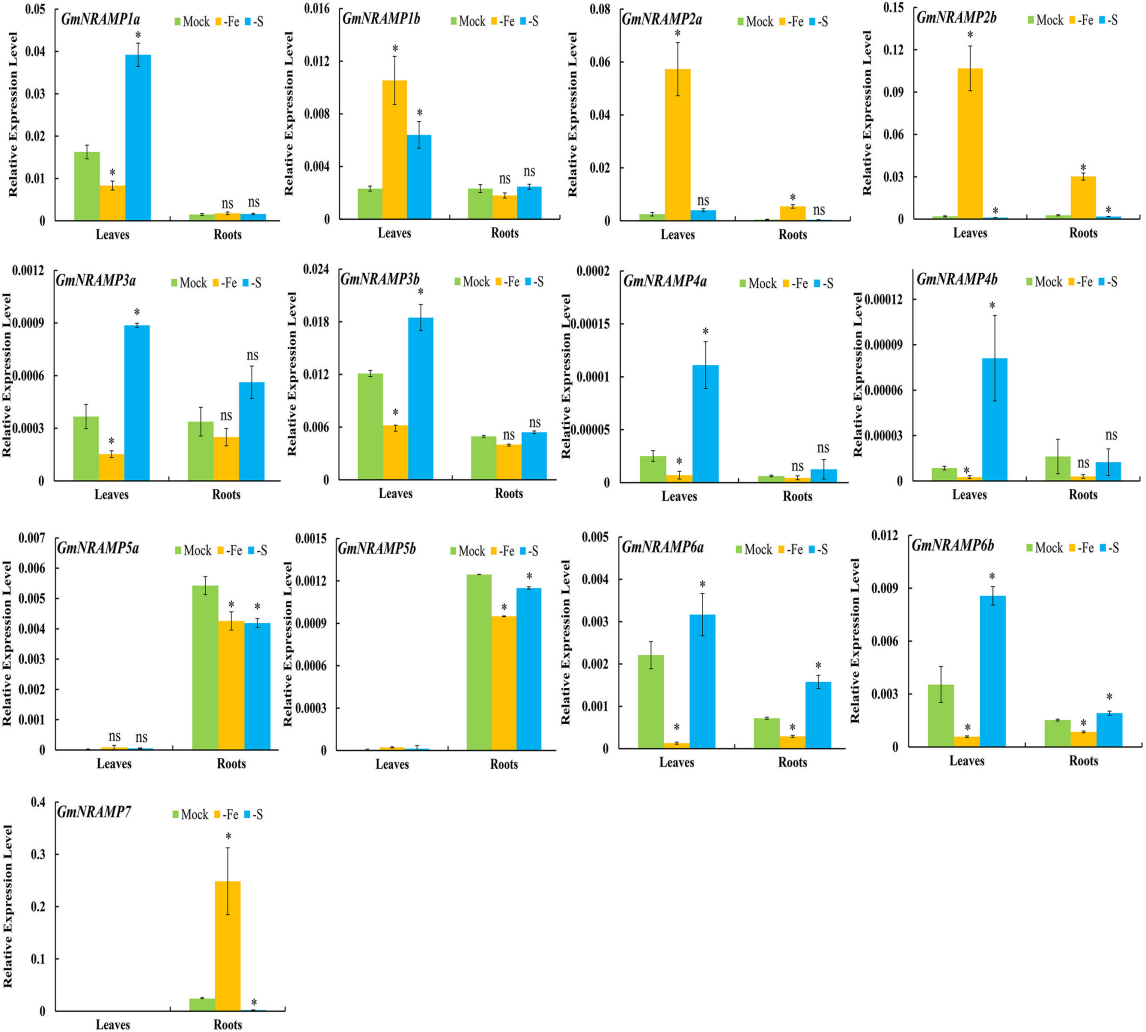
Expression of *GmNRAMPs* in response to Fe or S deficiency. Ten-day-old soybean seedlings were subjected to Fe or S deficiency for 14 days. Leaves and roots were separately harvested for qRT-PCR analysis. Fold-changes of *GmNRAMP* gene expression were normalized against the reference gene *TefS1* using 2-^ΔCt^ values. Each bar is the mean of four biological replicates with standard error. “^*^” indicates significant differences between soybean without and with rhizobia inoculation in young leaves, stems, and roots in one-way analysis of variance, ^*^P < 0.05, ns: not significant.

To further investigate potential roles for soybean *NRAMP* genes in nutrient homeostasis, *GmNRAMPs* were tested for alterations in expression in response to Fe or S deficiency. Two known marker genes, *GmIRT* (for low Fe) and *GmSULTR1;2b* (for low S) (Qin et al., 2012; Ding et al., 2016), were also applied to confirm the Fe or S deficiency in this study (Figures S2D,E). As shown in Figures 6, **9** *GmNRAMP* genes were significantly down-regulated by Fe deficiency. The abundance of *GmNRAMP6a* was down-regulated in both Fe-deficient leaves and roots. *GmNRAMP*s *1a, 3a, 3b, 4a, 4b*, and *6b* were also down-regulated only in leaves, and *GmNRAMP*s *5a* and *5b* were also down-regulated only in roots. Four *NRAMP* genes responded to Fe starvation with enhanced expression levels. Among them, *GmNRAMP2a* and *GmNRAMP2b* exhibited increased expression both in leaves and roots, while *GmNRAMP7* expression was enhanced in roots, and *GmNRAMP1b* was up-regulated in leaves. Furthermore, except for *GmNRAMP2a* and *GmNRAMP5b*, all soybean *NRAMP* genes also responded to S deficiency (Figure 6). Interestingly, responses to S deficiency were opposite of those observed for Fe deficiency, with the exception of three *GmNRAMPs*. Specifically, *GmNRAMP5a* was down-regulated in both Fe- and S-deficient roots, *GmNRAMP1b* was up-regulated in both Fe- and S-deficient leaves, and *GmNRAMP2a*, responded to Fe, but not to S (Figure 6).

### Expression of *GmNRAMPs* in response to divalent metal toxicity stresses

**Figure 7:**
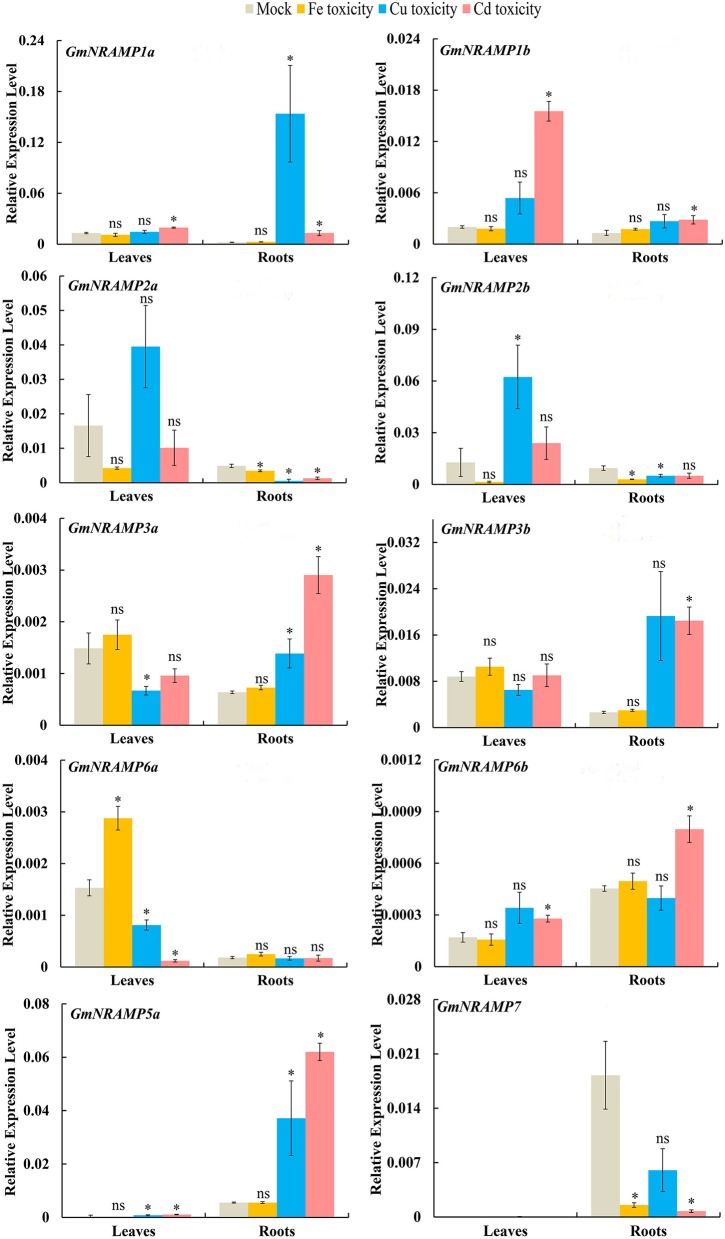
Expression of *GmNRAMPs* in response to different metal toxicity stresses. Ten-day-old seedlings were treated with excess Fe (1,000 μM EDTA-Fe), Cu (200 μM CuSO_4_·5H_2_O), and Cd (100 μM CdCl_2_) treatments for 24 h. Each bar is the mean of four biological replicates with standard error. “^*^” indicates effects of toxicity treatments relative to controls in one-way analysis of variance, ^*^P < 0.05, ns: not significant.

In order to evaluate the probable functions of *GmNRAMPs* in responses to divalent metal toxicity stresses, expression of these genes was also assayed by qRT-PCR in soybean seedlings exposed to excess supply of Fe, Cu, Cd, or Mn. Due to low abundances of *GmNRAMPs 4a, 4b*, and *5b* in leaves and roots under these treatments, only 10 *NRAMP* genes were analyzed in this experiment. In general, the expression of soybean *NRAMP* gene family members was most sensitive to Cd toxicity, followed by Cu toxicity. The expression of six *GmNRAMP* genes was greatly enhanced by excess Cd, with four of them were being up-regulated in both leaves and roots, and two of them were being enhanced in roots (Figure 7 and Table 3). While three *NRAMP* genes responded to Cd toxicity with reductions in expression levels (Figure 7 and Table 3). Under Cu toxicity stress, *GmNRAMP5a* was up-regulated in both leaves and roots, and *GmNRAMP1a* was up-regulated in roots, whereas the expressions of *GmNRAMP6a* and *GmNRAMP2a* were down-regulated in leaves and roots, respectively. In addition, two *NRAMP* genes, *GmNRAMP2b* and *GmNRAMP3a* exhibited opposite trends in soybean leaves and roots under excess Cu stress. In another, the expression of soybean *NRAMP* genes seemed less influenced by excess Fe supply in comparison to the other toxicity treatments or to Fe deficiency, with only three genes, *GmNRAMP2a, 2b*, and *7* being suppressed by Fe toxicity (Figure 7 and Table 3). Finally, in the Mn toxicity treatment, expression was altered for only four *NRAMP* genes, with three being up-regulated, while one was down-regulated (Figure S3 and Table 3).

**Table 3:** Summary of the response of soybean *NRAMP* genes to different divalent metal toxicities.

**Gene name**	**Fe toxicity**		**Cu toxicity**		**Cd toxicity**		**Mn toxicity**	
	**Leaves**	**Roots**	**Leaves**	**Roots**	**Leaves**	**Roots**	**Leaves**	**Roots**
*GmNRAMP1a*	−	−	−	↑	↑	↑	−	−
*GmNRAMP1b*	−	−	−	−	↑	↑	−	−
*GmNRAMP2a*	−	↓	−	↓	−	↓	↑	−
*GmNRAMP2b*	−	↓	↑	↓	−	−	↑	−
*GmNRAMP3a*	−	−	↓	↑	−	↑	↓	−
*GmNRAMP3b*	−	−	−	−	−	↑	−	−
*GmNRAMP5a*	−	−	↑	↑	↑	↑	−	↑
*GmNRAMP6a*	↑	−	↓	−	↓	−	−	−
*GmNRAMP6b*	−	−	−	−	↑	↑	−	−
*GmNRAMP7*	−	↓	−	−	−	↓	−	−

*Significant differences between treatment and control are marked as arrows. “↑” stands for up-regulated expression in response to the treatment, while “↓”stands for down-regulated expression in response to the treatment*.

### Subcellular localization of GmNRAMP proteins

**Figure 8:**
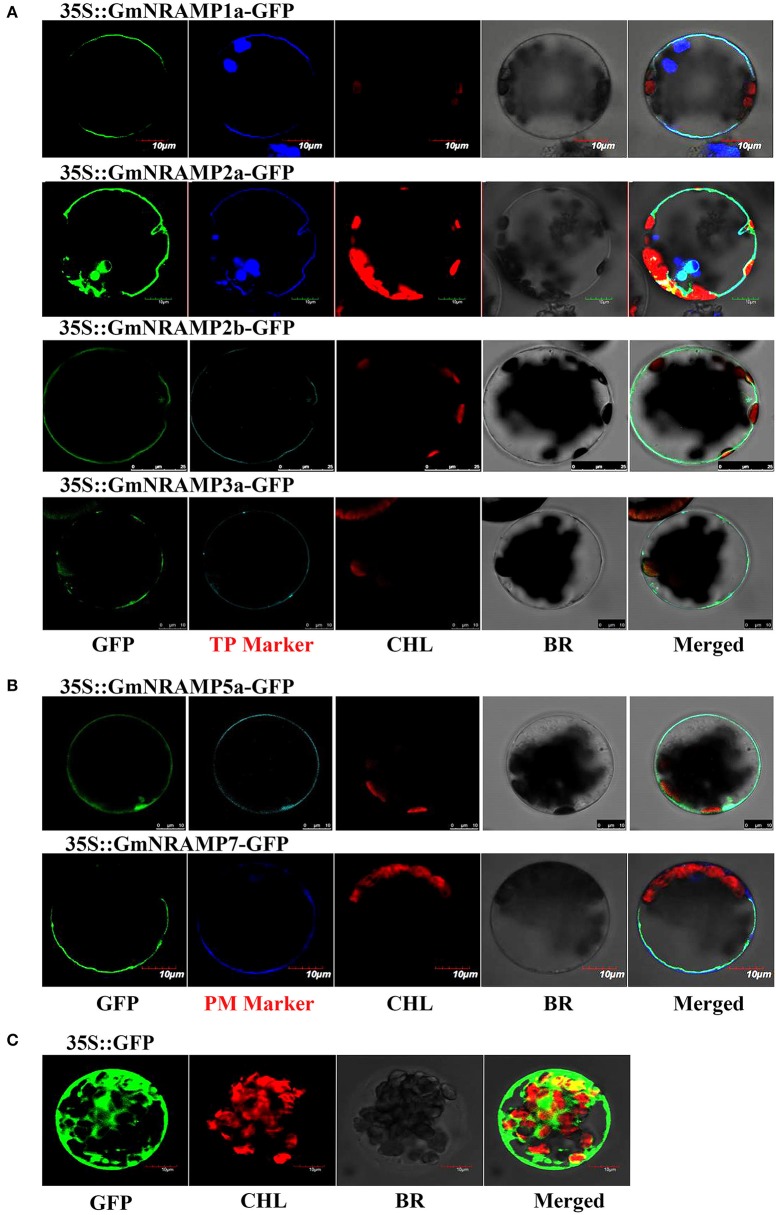
Subcellular localization of selected GmNRAMP proteins in *Arabidopsis thaliana* protoplasts. Subcellular localization of GmNRAMP1a, GmNRAMP2a, GmNRAMP2b, GmNRAMP3a, GmNRAMP5a and GmNRAMP7 was assessed in Arabidopsis protoplasts transiently transformed with 35S::GmNRAMP-GFP constructs. The tonoplast marker (TP Marker) AtTPK1 was included with 35S::GmNRAMP1a, 2a, 2b, 3a-GFP constructs **(A)**, and the plasma membrane marker (PM Marker) OsMCA1 was included with 35S::GmNRAMP5a and 7-GFP constructs **(B)**. 35S::GFP was included as a control **(C)**. GFP, green fluorescence signal; CHL, chloroplast; BR, bright field; Merged: GFP, CHL, Marker, and BR overlay.

To explore the subcellular localization of the GmNRAMP proteins, putative subcellular localizations were first identified by ProtComp analysis. As shown in Table 1, eight NRAMP proteins were predicted to localize to the vacuole, whereas the other five GmNRAMP proteins were predicted to target the plasma membrane. Subsequent to this computational analysis, subcellular localization for six selected GmNRAMPs proteins was empirically observed though transient expression of GFP: GmNRAMP fusions in Arabidopsis protoplasts containing the membrane marker OsMCA1 (Kurusu et al., 2012) or the tonoplast marker AtTPK1 (Voelker et al., 2006), with verification of these two specific localization makers conducted in co-transformed rice protoplasts (Figure S4). Microscopic observation revealed that the 35SGFP::GmNRAMP1a, 35SGFP::GmNRAMP2a, 35SGFP::GmNRAMP2b, and 35SGFP::GmNRAMP3a fusions were exclusively localized to the tonoplast, as evidenced by co-localization with the known tonoplast marker AtTPK1 (Figure 8A). The 35SGFP::GmNRAMP5a and 35SGFP::GmNRAMP7 fusions localized on the plasma membrane along with the membrane marker OsMCA1 (Figure 8B), and 35SGFP empty vector controls yielded whole cell fluorescence (Figure 8C). These results indicate that GmNRAMP proteins localize to different subcellular compartments. Furthermore, localization might be associated with specific biological functions in plant cells.

### Bioinformatic analysis of protein-protein interactions involving GmNRAMPs

To explore potential interactions among GmNRAMP members, protein-protein interaction analysis was conducted computationally in the Uniprot and STRING database. Predicted interacting proteins were nearly identical for 10 of the 13 GmNRAMP query proteins (network I in Figure S5A), including for GmNRAMP1a, GmNRAMP2a, and GmNRAMP4b to GmNRAMP7. All of the proteins predicted to interact with these 10 NRAMPs contain multicopper oxidase domains, and may be involved in the metabolism of ascorbate and aldarate (Table S5). In contrast to network I, GmNRAMP2b, GmNRAMP3a, and GmNRAMP3b were predicted to interact in another set of similar interaction networks as shown in Figure S5B. The putative networks for GmNRAMP3a, GmNRAMP3b, and GmNRAMP2b are very similar to each other, with seven identical interacting proteins labeled with a star in Figure S5B. These seven proteins include a Zn transporter, a vacuolar Fe transporter and a ferric reductase (Table S5). The interaction networks involving GmNRAMP3a and GmNRAMP3b share two proteins in common with network I as labeled with check marks, both of which contain multicopper oxidase domains. Meanwhile, GmNRMAP2b is predicted to interact with RBCS-1 (Ribulose bisphosphate carboxylase) and HDL56 (Transcription factor HEX, containing HOX, and HALZ domains). Interestingly, nodulin-21 and a nodulin-like protein were also found in the interaction networks of GmNRAMP2b, GmNRAMP3a, and GmNRAMP3b, suggesting potential functions for these GmNRAMPs in Fe or other metal ion transport in soybean nodules.

### Expression of *GmNRAMPs* in soybean nodules with rhizobia inoculation

**Figure 9:**
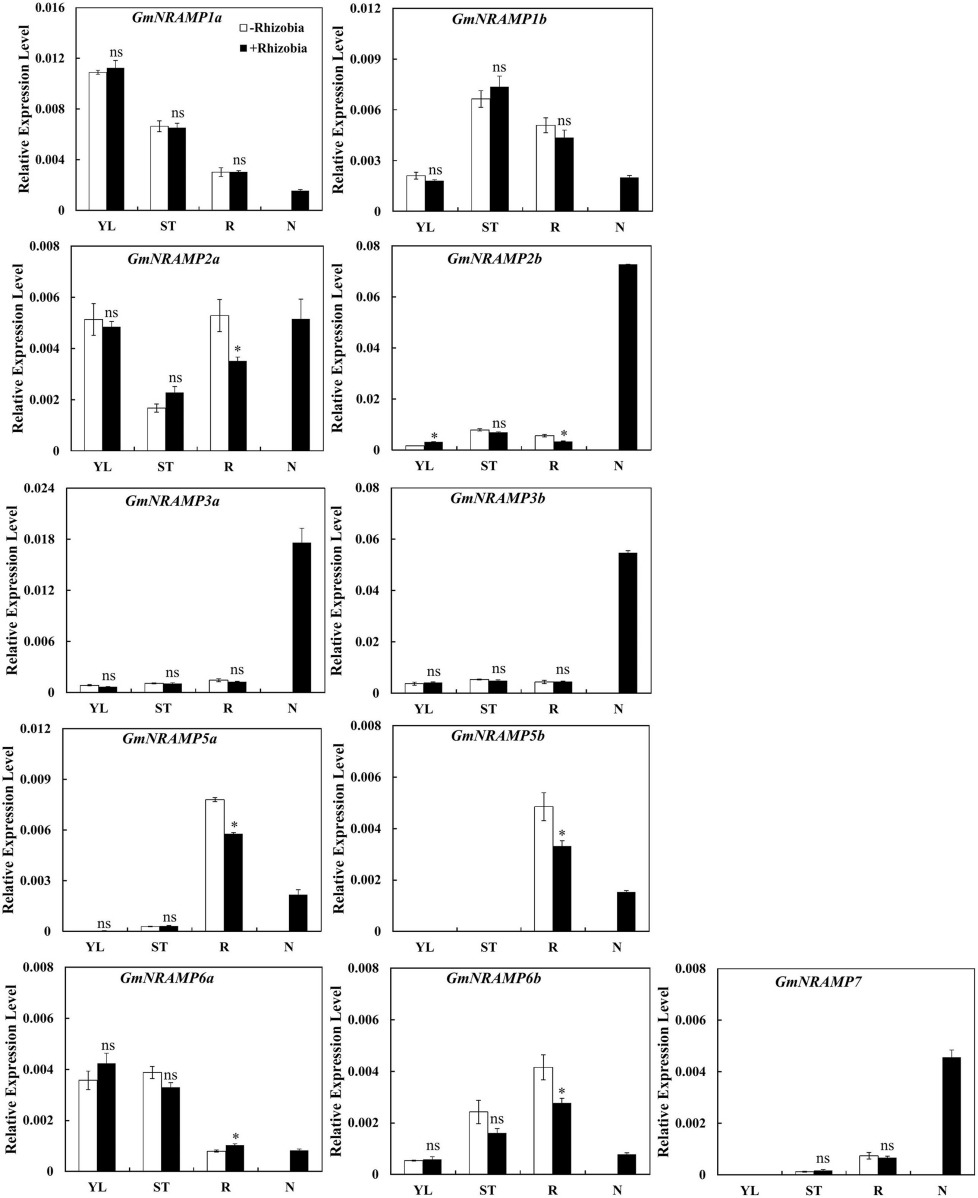
Expression of *GmNRAMPs* in response to rhizobia inoculation. Seven-day-old soybean seedlings were inoculated with rhizobial strain *Bradyrhizobium* sp. BXYD3 and then transplanted into low nitrogen (500 μM N added) nutrient solution. Various tissues including young leaves (YL), stems (ST), roots (R), and nodules (N) were separately collected 30 days after inoculation. Relative expression levels were obtained by real-time PCR normalized against the reference gene *TefS1*. Each bar is the mean of four biological replicates with standard error. “^*^” indicates significant differences between soybean without and with rhizobia inoculation in young leaves, stems, and roots in one-way analysis of variance, ^*^P < 0.05.

In order to determine whether *GmNRAMP* genes function in soybean nodules, the expression of *GmNRAMP*s were tested in different tissues of soybean 30 days after rhizobia inoculation. Due to low expression of *GmNRAMP4a* and *GmNRAMP4b* under low N conditions, only 11 *GmNRAMP* genes were evaluated in this experiment. As shown in Figure 9, expression of *GmNRAMP1a, GmNRAMP1b, GmNRAMP6a*, and *GmNRAMP6b* were lower in soybean nodules than in other tissues upon inoculation with rhizobia. Compared with non-inoculated soybean, expressions of *GmNRAMP*s 2a, 2b, 5a, 5b, and 6b were significantly reduced in soybean roots after inoculation with rhizobia (Figure 9), during which time expression in nodules ramped up considerably. Moreover, under rhizobial-inoculation conditions, expression of *GmNRAMP2b, GmNRAMP3a, GmNRAMP3b*, and *GmNRAMP7*also scaled up in soybean nodules, as indicated by the respective 21.66-, 13.96-, 11.96-, and 6.8-fold differences compared to expression in soybean roots (Figure 9). Higher expression of *GmNRAMP2b, GmNRAMP3a, and GmNRAMP3b* in nodules compared to other *GmNRAMP*s suggests that these genes might be important for the transport of Fe or other metal ions in soybean nodules.

## Discussion

NRAMP proteins are exist in a wide range of bacteria, animals and plants, to date, the *NRAMP* gene family has been reported in a number of plant species, but information on this family is limited for soybean, the most important cultivated legume. In this study, the soybean genome was comprehensively searched for *NRAMP* genes, which resulted in the identification of 13 putative *GmNRAMP* genes. Phylogenetic analysis clustered these 13 NRAMP proteins into two distinct subfamilies (Figure 1A). Interestingly, 12 of the 13 GmNRAMP proteins further clustered into six branches of paired proteins (Figure 1A and Table 2). These homologous pairs of GmNRAMPs might be the products of duplication events in soybean evolutionary history (Figure 1B). Altogether, the combination of homologous pairs and two subfamilies based on structural similarities guided the phylogenetically based naming of GmNAMPs employed herein. In Arabidopsis, the analysis of six *NRAMP* proteins has also revealed two subfamilies (Thomine et al., 2000; Mäser et al., 2001). Similarly to the current findings for soybean *NRAMP* genes, *AtNRAMP2* through 5 in subfamily I have 2–3 introns, while *AtNRAMP1* and *AtNRAMP6* in the other subfamily have 10 and 12 introns (data not shown in paper). On the other hand, unlike *AtNRAMP1* and *AtNRAMP6*, two soybean *NRAMP* genes in Subfamily II have an intron located on the 5′ UTR (Figure 1A). Previous studies have associated the presence of an intron within the 5′ UTR with enhanced RNA and protein accumulation (Rethmeier et al., 1997; Chung et al., 2006). In the current study, the two *GmNRAMPs, GmNRAMP6a* and *GmNRAMP6b*, which containing an intron within the 5′ UTR, were not among the most highly expressed genes in any of the tested tissues or conditions. Whether these 5′ UTR introns play roles in the regulation of *GmNRAMP6a* or *GmNRAMP6b* remains to be determined. Next, phylogenetic analysis of the 13 GmNRAMP proteins identified here, along with NRAMP protein sequences from other plant species (Figure 3), revealed that NRAMP proteins representing both subfamilies are common in both dicots and monocots. This suggests that the NRAMP family fulfills similar and basic functions in widely divergent plant species. Interestingly, soybean NRAMP proteins in the same subfamily were predicted and confirmed to have similar subcellular localizations (Figure 8 and Table 1). Such compartmentalization is likely related to specific functions, namely, uptake of metals on the plasma membrane, or release from vacuolar stores on the tonoplast as previously reported in Arabidopsis and rice. Specifically, AtNRAMP3 and AtNRAMP4 have been reported to be localized in Arabidopsis vacuoles, which is in consistent with roles in the release of metals from vacuolar stores (Lanquar et al., 2005). Meanwhile, OsNRAMP1, OsNRAMP3, and OsNRAMP5 have been identified as plasma membrane-localized proteins in rice participate in metal uptake (Takahashi et al., 2011; Sasaki et al., 2012; Yamaji et al., 2013). Phylogenetic analysis with these known NRAMP proteins implies potentially similar functions for soybean NRAMP proteins.

*NRAMP* genes functions in metal ions uptake, especially Fe, are widely found in mice, humans and plants, particularly under Fe-deficiency conditions. Although the *IRT/FRO* system seems to be a major component of Fe-uptake system in the non-graminaceous plants, several previous researches revealed that other genes could be involved in this process, such as *NRAMP* genes. In Arabidopsis, *AtNRAMP1* can complement the Fe uptake mutant of yeast, and appears to be expressed preferentially in Fe-deficient roots, indicating its role in Fe uptake and transport (Curie et al., 2000). In soybean, 13 *GmNRAMP* genes were significantly affected by Fe deficiency in leaves or roots of soybean (Figure 6), indicating that *GmNRAMP* genes also are involved in Fe nutrition. For root-expressed *GmNRAMP* genes, *GmNRAMP7* was remarkably up-regulated by Fe deficiency in roots (Figure 6), combining with its plasma membrane localization (Figure 8), implying that *GmNRAMP7* might participate in the acquisition of Fe on the plasma membrane in root cell, perhaps also coupling with *IRT* and *FRO* as integral part of plant root cell Fe uptake machinery under Fe starvation stress. However, this hypothesis would be further investigated by the tissue and cell specific localization of GmNRAMP7 by GUS staining and GFP fluorescence in future trails. Besides, previous study showed that overexpression of *AtNRAMP1* leads to an increased resistance to toxic Fe level (Curie et al., 2000), indicating it may participates in Fe remobilization under Fe deficiency, despite this function was not consistent with its plasma membrane localization. However, it is uncertain that this kinds of gene always going to be present in cell membranes? It's interesting to speculate that if *AtNRAMP1 or GmNRAMP7* was not always present in cell membrane, whether it might performed potential functions in Fe or other metals transport in root cell, for example, remobilization of Fe stored in organelles under Fe-deficient conditions as *MxNRAMP1* which mainly exists in the plasma membrane and vesicles (Pan et al., 2015), or regulate homeostasis of free ions which might induced by Fe starvation, mediate sequestration of free ions into a cellular compartment, such as plastid or vacuole, all of these speculations also need further study.

Beyond functioning in Fe transport, *NRAMP* genes have been also demonstrated to perform wide ranging transport activities for divalent transition metals, including Mn^2+^, Fe^2+^, Co^2+^, Ni^2+^, Cu^2+^, and Zn^2+^ (Illing et al., 2012). Recently, several studies have reported the substantial role of *NRAMP* family members in Mn uptake. As shown in Figure 3, four NRAMP proteins, GmNRAMP5a, GmNRAMP5b, GmNRAMP6a, and GmNRAMP6b cluster together into a small phylogenetic branch with AtNRAMP1, a plasma membrane localized high-affinity Mn transporter (Cailliatte et al., 2010). This small branch is also closely related to OsNRAMP3, which is a known Mn transporter that is involved in shoot Mn distribution, and is constitutively expressed in nodes, stems and panicles, where it mediates adaptation of rice to a wide change of external Mn conditions (Yamaji et al., 2013; Yang et al., 2014). Interestingly, one soybean NRAMP protein, GmNRAMP5a, clustered in the phylogenetic tree with AtNRAMP1 and OsNRAMP3 (Figure 3), and was also affected by Mn as indicated by the significant increase in its expression in response to Mn toxicity (Figure S3 and Table 3), suggesting it is probably important for Mn homeostasis in soybean. In addition, *NRAMP* genes also seem to affect the intracellular remobilization of divalent toxic heavy metals, such as Cd (Cailliatte et al., 2009), which might contribute to increase plant tolerance to heavy metal toxicity. In this study, the responses of *GmNRAM*P genes to different divalent metal toxicities were also investigated (Figure 7 and Table 3). As expected, the remarkable changes in expression were observed for most *GmNRAMPs* under excess Cd treatment, suggesting that these genes might contribute to Cd tolerance in soybean plants under Cd toxicity stress. Interestingly, among 13 soybean *NRAMP* genes, the expressions of *GmNRAMP1a, GmNRAMP3a, GmNRAMP5a* in roots were both dramatically enhanced by Cu and Cd toxicities (Figure 7 and Table 3), furthermore, the expression of *GmNRAMP5a* in roots were significantly increased by Cu, Cd, and Mn toxicities (Figure 7, Figure S3 and Table 3). In consequence, we propose that soybean *NRAMP* genes widely participated in Fe, Mn, Cu, and Cd transport, might be involved in the uptake and homeostasis regulation of these metal ions.

Moreover, considering the fact that macronutrient deficiencies often exist in field conditions, the responses of soybean *NRAMP* genes to N, P, and K deficiencies were also evaluated in this study. Most *GmNRAMP* responses to N deficiency involved down regulation, while, in contrast, a majority of *GmNRAMP* genes were remarkably enhanced by P deficiency (Figure 5). This enhancement of *NRAMP* gene expression under low P conditions is similar to patterns observed for soybean Fe-S assembly genes (Qin et al., 2015). A considerable amount of research has outlined interactions between P and Fe response pathways in plants. Phosphorus deficiency can increase carbon flux through glycolysis for the synthesis of organic acids, change lipid metabolism, and affect the abundance of genes involved in Fe and Zn metabolism (Wasaki et al., 2003; Zheng et al., 2009). Notably, increased Fe concentrations are often observed in P-deficient plants (Misson et al., 2005; Hirsch et al., 2006). The correlated Fe and P responses described in the current and previous studies indicate the existence of linkages between P and Fe metabolism through one or more pathways, including those involved with transport, homeostasis and accumulation. In previous work, P deficiency affected Fe storage, as indicated by the accumulation of Fe associated with ferritin in chloroplasts (Hirsch et al., 2006). More recently, cross-talk between P and Fe homeostasis pathways has been demonstrated in Arabidopsis, where the ferritin gene, *AtFer1*, is regulated by the phosphate starvation response transcription factor *AtPHR1* (Bournier et al., 2013). In contrast, P deficiency does not appear to affect Arabidopsis genes encoding strategy I Fe-uptake system proteins, such as *IRT1* (Hirsch et al., 2006). Whether the expression of other Fe uptake transporters is triggered in P-deficient Arabidopsis remains unknown. In this study, significant changes in the expression of *GmNRAMP* genes in P-deficient soybean plants reinforces the idea that plant P and Fe response pathways are linked and interact, and further imply that low P impacts Fe uptake and homeostasis through the activities of *GmNRAMP* transport systems. However, comprehensive elucidation of linkages between P and Fe metabolic pathways and the underlying mechanisms requires further investigation. Interactions between P and Fe pathways in plants has also been observed in jasmonate ZIM domain (*JAZ*) genes, which act as transcriptional repressors of jasmonate-responsive genes, as indicated by highly altered expression of such genes in rice and chickpea experiencing mineral nutrient deficiency (Singh et al., 2015). What's more, observations of the involvement of *JAZ* genes in nutrient deficiency responses contribute to further understanding of the roles jasmonates play in regulating nutrient deficiency response adaptations. It is noteworthy that several *cis*-acting elements involved in hormone-responsiveness were found in the putative promoter regions of soybean *NRAMP* genes (Table S4), but the regulatory mechanisms remain unclear. Whether NRAMP involvement in the uptake or homeostasis of metal ions is related to hormone-regulated morphological and physiological responses to nutrient and toxin status requires further study. One final interaction relevant here is the coordination of Fe with S in Fe-S proteins, which are the biggest Fe sink in plants. Unsurprisingly, therefore, interplay between Fe and S has been noted in recent years (Zuchi et al., 2015). Interestingly, most soybean *NRAMP* genes displayed contrasting responses to Fe and S deficiencies (Figure 6). Since Fe-S clusters are significant Fe sinks within cells, the deprivation of Fe or S will depress the synthesis of Fe-S clusters. Changes in *GmNRAMP* expression in response to Fe- or S-deficiency suggest links between these two stress responses. More definitive demonstrations of whether *NRAMP* genes are involved in coordinated regulation of Fe and S homeostasis, as well as, the synthesis of Fe-S clusters requires further investigation.

It is worth mentioned that several soybean NRAMP proteins were not only found to interact with metal ion transporters, such as the Zn/Fe transporter, but several were also observed in interactions with nodulin and nodulin-like protein (Figure S5 and Table S5). The high abundance of *GmNRAMP2b, GmNRAMP3a*, and *GmNRAMP3b* in soybean nodules confirmed a predicted interaction network (Figure S5B) and suggested possible functions for these *GmNRAMP* genes in soybean-rhizobia symbiosis. Besides these three *NRAMP* genes, *GmNRAMP2a* and *GmNRMAP7* were also detected in nodules at relatively high expression levels (Figure 9). However, these two proteins were not predicted to interact with nodulation-related proteins. The expression patterns of *GmNRAMP2a* in various soybean tissues subsequent to rhizobia inoculation did not correspond to those of its homolog *GmNRAMP2b*, which might be due to the evolutionary divergence. As shown in Figure 3, the nodule-expressed subfamily II gene *GmNRAMP7* has diverged from the other four nodule-expressed *NRAMP* genes. GmNRAMP7 is the only soybean NRAMP protein in a small branch with several monocot NRAMPs (including OsNRAMP1, OsNRAMP5, and HvNRAMP5), which are mainly expressed in roots (Sasaki et al., 2012; Wu et al., 2016). This is consistent with the tissue-specific expression of *GmNRAMP7* (Figure 4). It also clusters with AhNRAMP1, a Fe transporter proven to be involved in Fe acquisition (Xiong et al., 2012). Most notably, GmNRAMP7 also clusters closely with MtNRAMP1, which has been reported to be most highly expressed in roots and nodules, as well as, the main transporter responsible for Fe uptake in nodule cells (Tejada-Jiménez et al., 2015). Taken together, the results herein imply that *GmNRAMP7* might function in Fe uptake during symbiotic nitrogen fixation. Finally, GmNRAMP7 is predicted to be localized to the plasma membrane, while the four other nodule-expressed *NRAMP* genes were predicted to localize on the tonoplast. Any specific roles for these genes in the regulation of metal ions homeostasis requires further study.

## Conclusion

In the present study, 13 *NRAMP* genes were identified from the soybean genome and, subsequently, named according to the phylogenetic relationships inferred among them. Expression profiles of *GmNRAMP* genes varied among soybean tissues and in response to a series of nutrient stresses, which suggests that *GmNRAMPs* perform a range of functions in specific tissues throughout growth and development. Furthermore, this gene family also likely participates in crosstalk among different nutrient stress pathways. Then the subcellular localization analysis in Arabidopsis protoplasts confirmed the tonoplast or plasma membrane localization of soybean NRMAP proteins. Taken together, the results reported here comprise a systematic genome-wide analysis of the soybean *NRAMP* gene family. These results supply basic and important information for understanding the putative functions of *NRAMP* genes in soybean. Moreover, protein-protein interaction network and qRT-PCR analysis in rhizobia-infected soybean further revealed that 3 NRAMP proteins putatively interact with nodulin-like proteins and are markedly up-regulated in soybean nodules. These results suggest potential functions for a subset of GmNRAMP proteins in the uptake of Fe or other metals, as well as, regulation of homeostasis in soybean nodules. Overall, this study provides valuable information for further functional studies on the biological roles of *NRAMP* genes in soybean. Plus, this report provides a basis for further understanding crosstalk between different nutrient response pathways in plants.

## Author contributions

LQ and XL conceived the study, analyzed the data, and drafted the manuscript. LQ, XH, and LX cultivated the soybean materials and collected the soybean samples. PH, LC, and YL extracted RNA and performed the qRT-PCR experiments. LC designed the vectors for subcellular localization. TW conducted the statistical analyses of raw data. HL and TW revised the manuscript. All authors read and approved the final manuscript.

### Conflict of interest statement

The authors declare that the research was conducted in the absence of any commercial or financial relationships that could be construed as a potential conflict of interest.
