# Supplementary material for: Genome-Wide Identification and Expression Analysis of NRAMP Family Genes in Soybean (Glycine Max L.)
Source: Front Plant Sci. 2017 Aug 18;8:1436. doi: 10.3389/fpls.2017.01436 (PMC5563376; doi:10.3389/fpls.2017.01436)
Supplement: Supplementary file 1 [file Table1.DOC]

**Supplementary Table S1.** **Soybean *NRAMP*** **genes and gene-specific primers used for quantitative real-time PCR experiments.**

| *GmPTs* | Sequence (5’ to 3’) | Amplicon size |
| --- | --- | --- |
| *GmNRAMP1a* | F:TCAAGAAGGGTTGACCCCAG  R:CTCCTGAAGGTACTGCCCTG | 195 |
| *GmNRAMP1b* | F:TCAAGGCAGGTTGATCCCAG  R:TCTTGAAGGTACTGCCCTGC | 194 |
| *GmNRAMP2a* | F:GCCACTATTAGCGGACTCGT  R:CGGGATCCAGAAACGCTATG | 200 |
| *GmNRAMP2b* | F:GATAGTGGTGGTCGGAGTCG  R:AGGTCCCCCTCTAAGTTTCCA | 150 |
| *GmNRAMP3a* | F:CATCATGGGAGGAAGAGGTG  R:CCAGTGAACAGCCACAGCTT | 130 |
| *GmNRAMP3b* | F:CTACTATTCAATTGAATCGGCG  R:CTCTTCAAGATACTGCCCTGC | 145 |
| *GmNRAMP4a* | F:GTGGTGTAAGGGAAGGGTCC  R:ACCAAAGCAACGTGTACCCT | 162 |
| *GmNRAMP4b* | F:ACTGAGGGCTTTCTTAATCTCAA  R:CATTAAGCCATTCATTCATGGT | 145 |
| *GmNRAMP5a* | F:TTCATCATCTGGGCAACCTCA  R:ACTAGAAATCCAGGTCCCACG | 159 |
| *GmNRAMP5b* | F:GATCGCATGTGACTCGGACT  R:TTTGAGAAGCTCCGGCTACC | 151 |
| *GmNRAMP6a* | F:CAGGGAATCAGAGAGGCTTGC  R:TCCAAATCCTGACAGCTCATCT | 158 |
| *GmNRAMP6b* | F:AAGGGTCTTTTTGTGCCAGGA  R:AAACCTGCAAGCCTCCCTAA | 165 |
| *GmNRAMP7* | F:CTGGAACCTATGCAGGGCAA  R:TGATGATAAGCCGACCAGCC | 156 |
| *TefS1* | F:TGCAAAGGAGGCTGCTAACT | 200 |
|  | R:CAGCATCACCGTTCTTCAAA |  |
