# Supplementary material for: Genome-Wide Identification and Expression Analysis of NRAMP Family Genes in Soybean (Glycine Max L.)
Source: Front Plant Sci. 2017 Aug 18;8:1436. doi: 10.3389/fpls.2017.01436 (PMC5563376; doi:10.3389/fpls.2017.01436)
Supplement: Supplementary file 2 [file Table2.DOC]

**Supplementary Table S2.** **Marker genes used in the nutrient deficiency experiment and gene-specific primers used for quantitative real-time PCR.**

| *Gene name* | Sequence (5’ to 3’) | Amplicon size | |
| --- | --- | --- | --- |
| *GmNiR* | F:TAGCCGACGAATACGCCAC  R:ATCTCCCCTTCAAGAGAGGC | | 120 |
| *GmPLDZ* | F:TGGTGCTTTCTGTGTGAAGC  R:GCTTTTCAGGATCCCCACCA | | 136 |
| *GmHAK* | F:CTGATGAAGCCGAAAGACCCA  R:CTGGTAGGGATCGTGTGCAA | | 108 |
| *GmIRT* | F:TGGTGGGGACCAATTGGAgA  R:GCTGTGCTTCTTTGCCCTCA | | 98 |
| *GmSULTR1;2b* | F:AATATGCACTCTACACCAGTTTTGTGT  R:GGGTCCTATGGCAATATC | | 80 |
