# Supplementary material for: Genome-Wide Identification and Expression Analysis of NRAMP Family Genes in Soybean (Glycine Max L.)
Source: Front Plant Sci. 2017 Aug 18;8:1436. doi: 10.3389/fpls.2017.01436 (PMC5563376; doi:10.3389/fpls.2017.01436)
Supplement: Supplementary file 3 [file Table3.DOCX]

**Supplementary Table S3.** **List of primers used in vector constructions for subcellular localization of six selected soybean NRAMP proteins.**

| *GmNRAMPs* | Sequence (5’ to 3’) |
| --- | --- |
| *GmNRAMP1a* | F: GGGGacaagtttgtacaaaaaagcaggctTCATGCCTCCTCAAGAGACCCAAC  R:GGGGaccactttgtacaagaaagctgggtCATTCACTGTGGTAGCAACTGTCTTTG |
| *GmNRAMP2a* | F: GGGGacaagtttgtacaaaaaagcaggctTCATGTCTCGGAGCCACCAAGAG  R: GGGGaccactttgtacaagaaagctgggtCACCCGCTGAGTGAGCAATTGTC |
| *GmNRAMP2b* | F: GGGGacaagtttgtacaaaaaagcaggctTCATGTCTGGGAGCCACCAAGA  R: GGGGaccactttgtacaagaaagctgggtCACCCTCTGAATGAGCAATTGTCTT |
| *GmNRAMP3a* | F: GGGGacaagtttgtacaaaaaagcaggctTCATGAGTGCTCCGTCGCGAGAA  R: GGGGaccactttgtacaagaaagctgggtCATTCCCACAAGAAGAAAATCCTTTG |
| *GmNRAMP5a* | F: GGGGacaagtttgtacaaaaaagcaggctTCATGGCTAGCCTGACACAGCATCAA  R: GGGGaccactttgtacaagaaagctgggtCGTTGTGGTAGTGGGATATCAGCCA |
| *GmNRAMP7* | F:GGGGacaagtttgtacaaaaaagcaggctTCATGACTGTGACAGGTTCATCATCTG  R: GGGGaccactttgtacaagaaagctgggtCATCAATTTCTGCTGGACTCCTTT |
