# Supplementary material for: Genome-Wide Identification and Expression Analysis of NRAMP Family Genes in Soybean (Glycine Max L.)
Source: Front Plant Sci. 2017 Aug 18;8:1436. doi: 10.3389/fpls.2017.01436 (PMC5563376; doi:10.3389/fpls.2017.01436)
Supplement: Supplementary file 5 [file Table5.DOCX]

**Supplementary Table S5. Proteins putatively interacting with soybean NRAMP family proteins**

| **Nodes in interaction network** | **Protein locus** | | **Annotation** | | |
| --- | --- | --- | --- | --- | --- |
| **GmNRAMP1a, GmNRAMP1b , GmNRAMP2a, GmNRAMP4a to GmNRAMP7** | | | | | |
| GLYMA20G33470.2 | | Glyma.20G192900 | | uncharacterized protein | |
| GLYMA20G33460.1 | | Glyma.20G192800 | | monocopper oxidase-like protein SKU5-like | |
| GLYMA20G12150.1 | | Glyma.20G051900 | | multicopper oxidases | |
| GLYMA19G07512.1 | | Glyma.19G054000 | | multicopper oxidases | |
| GLYMA20G31270.1 | | Glyma.20G172600 | | laccase | |
| GLYMA20G03030.1 | | Glyma.20G025200 | | L-ascorbate oxidase | |
| GLYMA20G33101.1 | | Glyma.20G189800 | | laccase | |
| AO | | Glyma.20G051700 | | L-ascorbate oxidase | |
| GLYMA20G12230.2 | | Glyma.20G051600 | | ascorbate oxidase-like protein-related | |
| GLYMA20G31280.1 | | Glyma.20G172700 | | laccase | |
| GLYMA20G12150.1 | | Glyma.20G051900 | | multicopper oxidases | |
| **GmNRAMP3a and GmNRAMP 3b** | | | | | |
| GLYMA08G08120.1 | | Glyma.08G076300 | | vacuolar iron transporter homolog 2.1 | |
| GLYMA15G13090.1 | | Glyma.15G124700 | | ferric reductase, NADH/NADPH oxidase and related proteins | |
| GLYMA06G05460.4 | | Glyma.06G052000 | | Fe2+/Zn2+ regulated transporter | |
| GLYMA14G37560.1 | | Glyma.14G196200 | | zinc transporter 6 | |
| GLYMA16G03770.1 | | Glyma.16G033800 | | ferric reductase, NADH/NADPH oxidase and related proteins | |
| GLYMA15G41620.1 | | Glyma.15G262800 | | zinc/iron transporter | |
| LOC547695 | | Glyma.05G121600 | | vacuolar iron transporter homolog 2.1 | |
| GLYMA20G33470.2 | | Glyma.20G192900 | | uncharacterized protein | |
| GLYMA20G33460.1 | | Glyma.20G192800 | | monocopper oxidase-like protein SKU5-like | |
| LOC547974 | |  | | nodulin-21 | |
| **GmNRAMP2b** | |  | |  |  |
| LOC732549 | | Glyma.10G276700 | | uncharacterized nodulin-like protein | |
| HDL56 | | Glyma.08G132800 | | transcription factor HEX, contains HOX and HALZ domains | |
| RBCS-1 | | Glyma.13G046200 | | ribulose-bisphosphate carboxylase small chain | |
