# Supplementary figures and images for: Genome-Wide Identification and Expression Analysis of NRAMP Family Genes in Soybean (Glycine Max L.)
Source: Front Plant Sci. 2017 Aug 18;8:1436. doi: 10.3389/fpls.2017.01436 (PMC5563376; doi:10.3389/fpls.2017.01436)

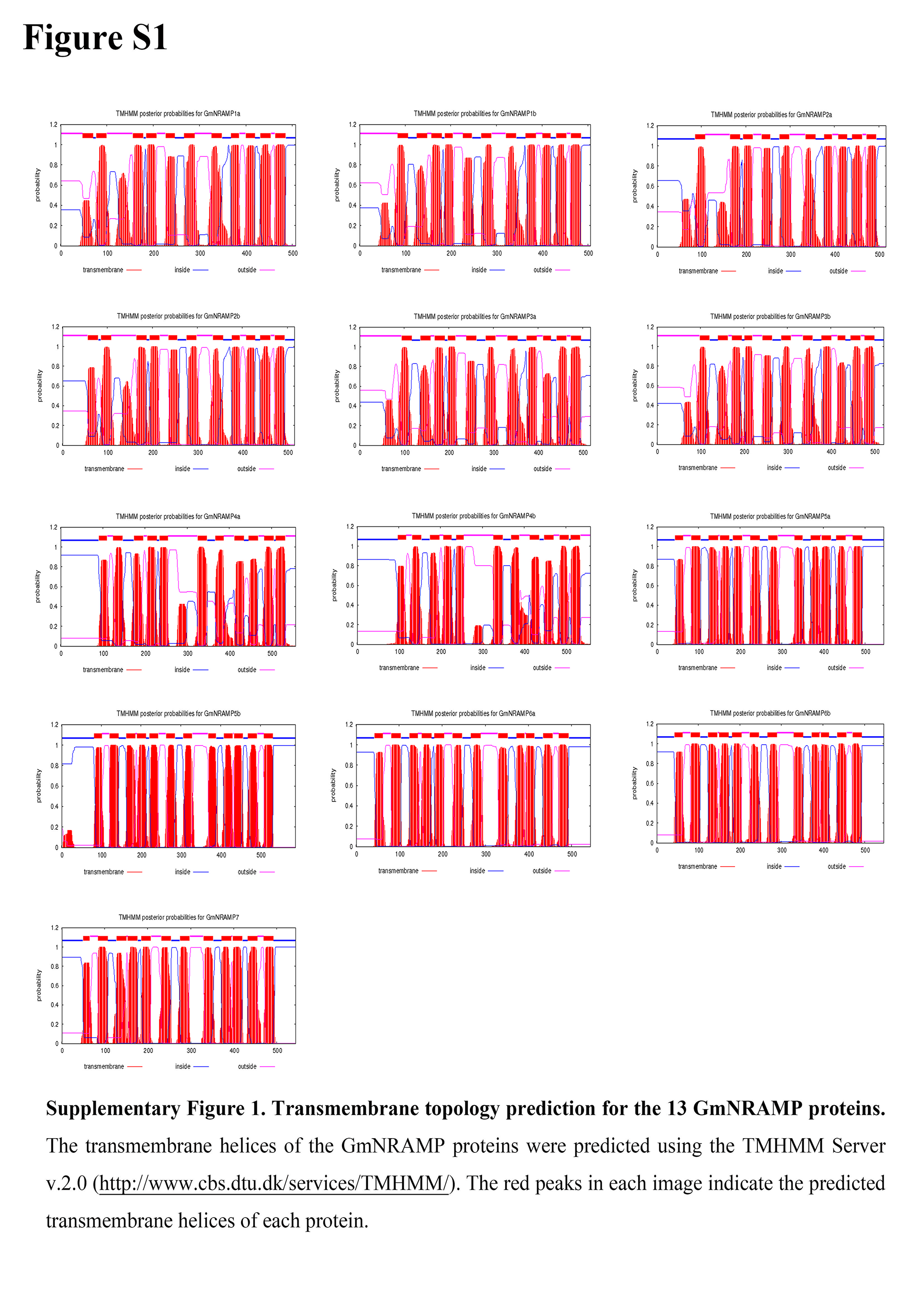

Supplement: Supplementary file 6 [file Image1.TIF]

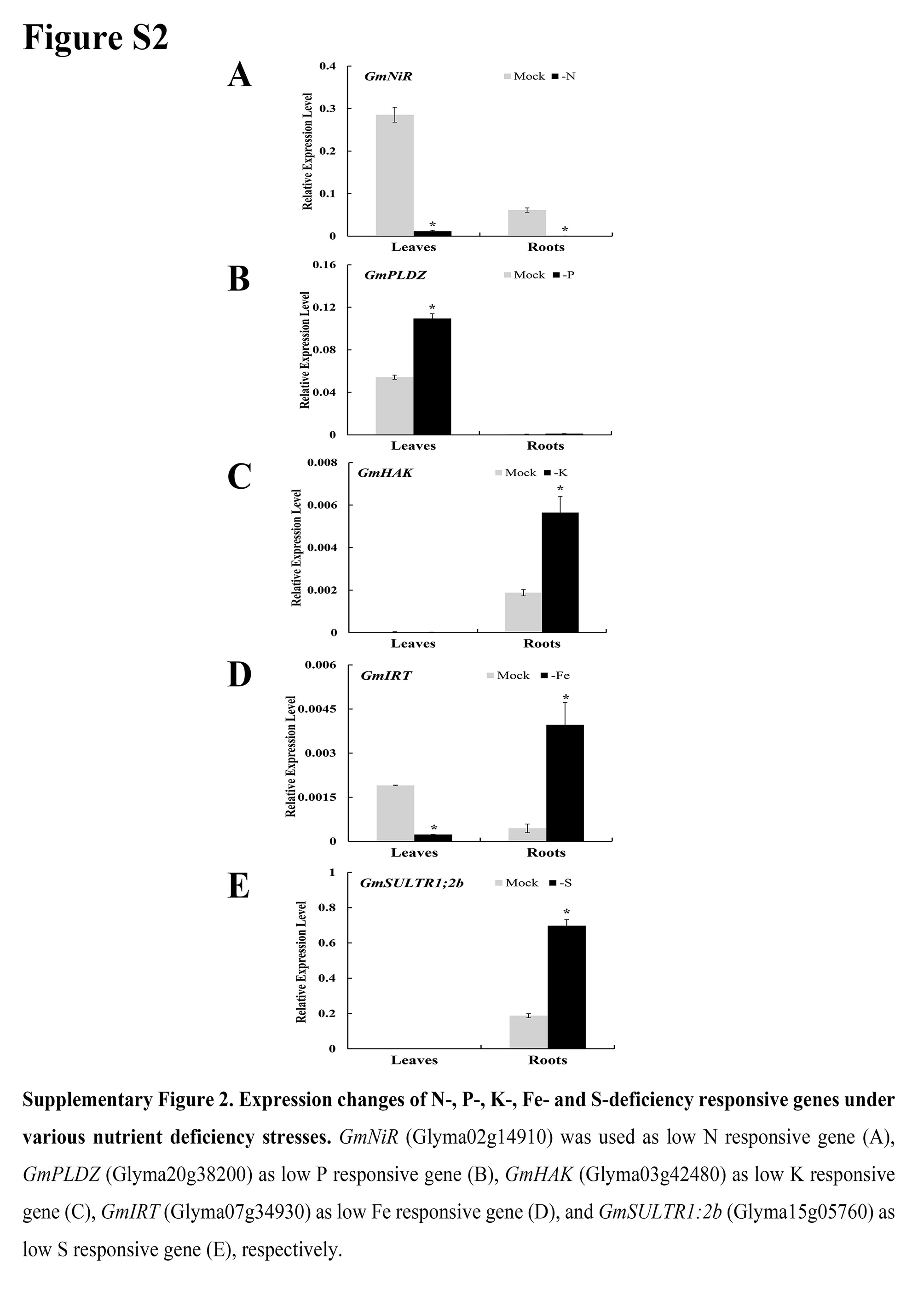

Supplement: Supplementary file 7 [file Image2.TIF]

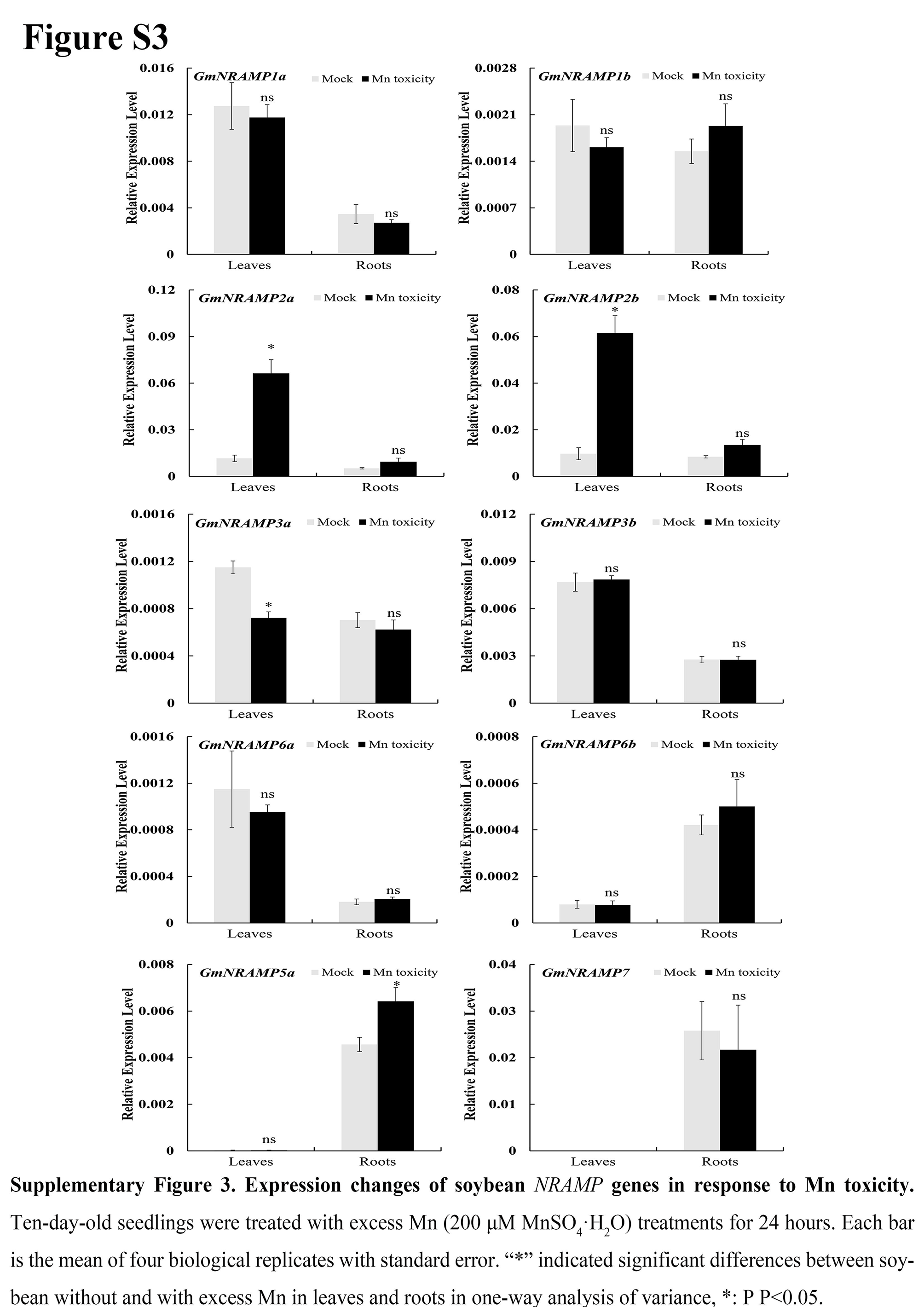

Supplement: Supplementary file 8 [file Image3.TIF]

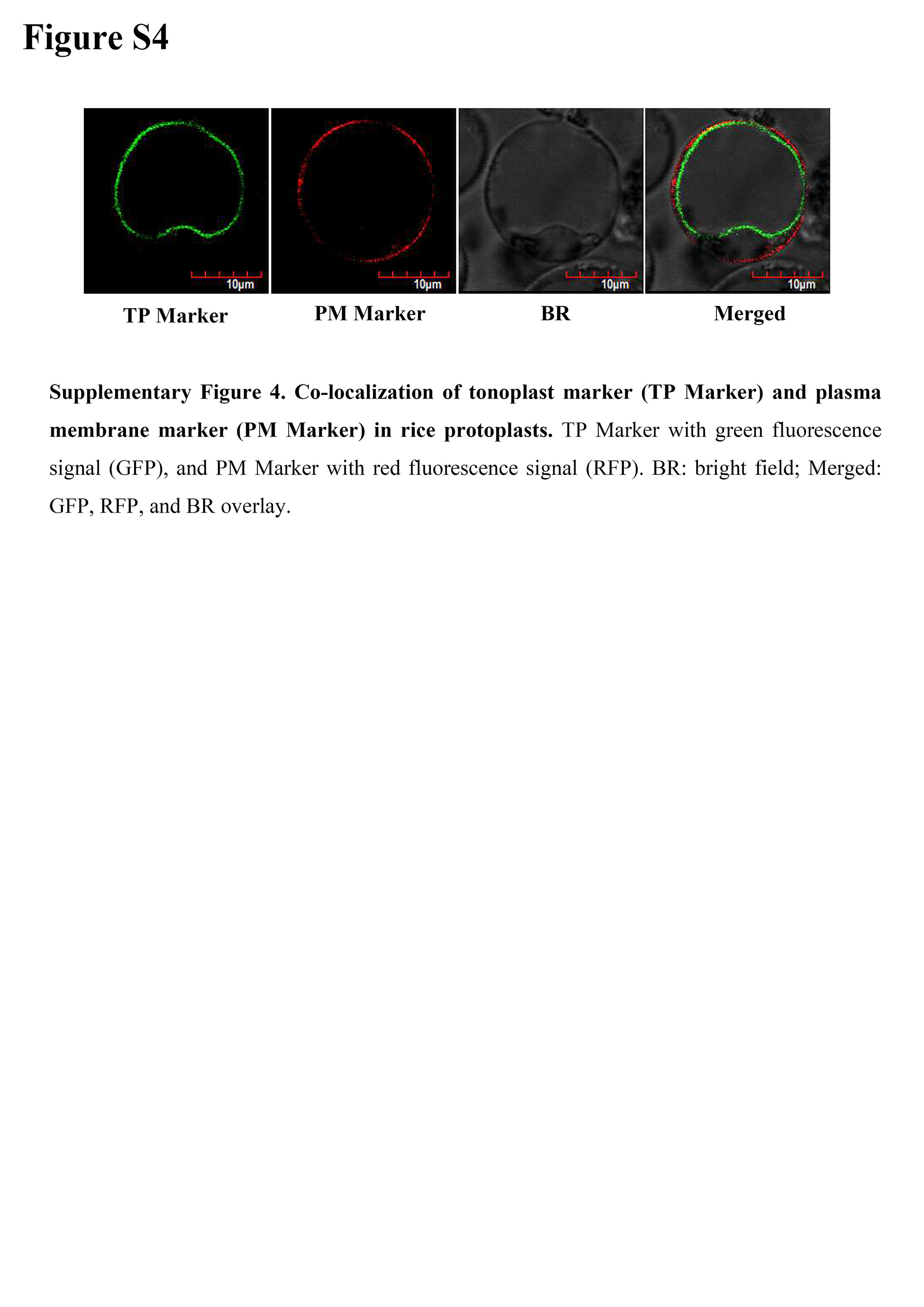

Supplement: Supplementary file 9 [file Image4.TIF]

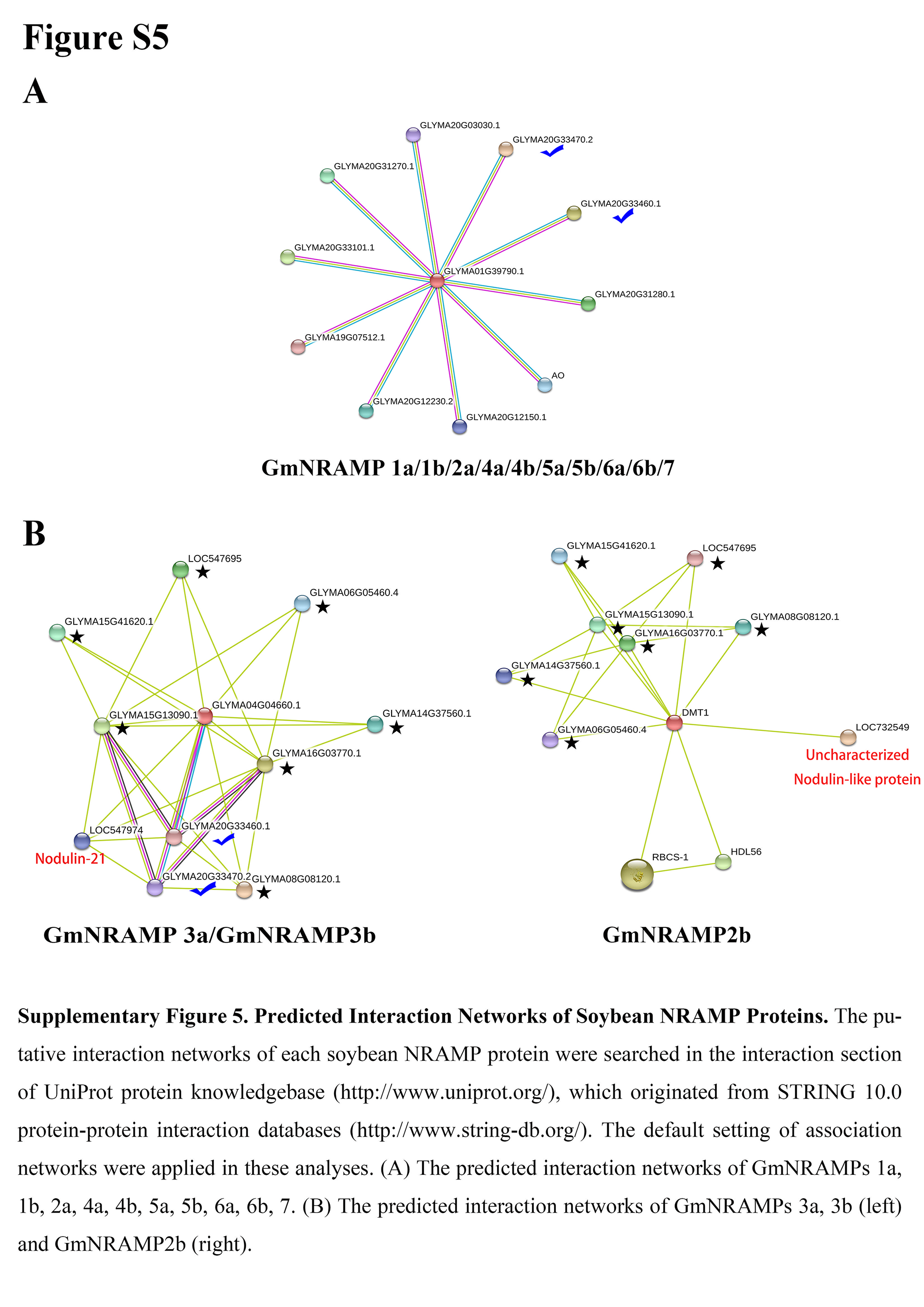

Supplement: Supplementary file 10 [file Image5.TIF]
